# Targeting PIM2 improves antitumor immunity through promoting effector function and persistence of CD8 T cells

**DOI:** 10.1172/JCI192928

**Published:** 2026-01-27

**Authors:** Yongxia Wu, Linlu Tian, Allison Pugel, Reza Alimohammadi, Qiao Cheng, Weiguo Cui, Michael I. Nishimura, Lauren E. Ball, Chien-Wei Lin, Shikhar Mehrotra, Andrew S. Kraft, Xue-Zhong Yu

**Affiliations:** 1 Department of Microbiology & Immunology and; 2 The Cancer Center, Medical College of Wisconsin, Milwaukee, Wisconsin, USA.; 3 School of Medicine, Northwestern University, Chicago, Illinois, USA.; 4 Department of Surgery, Stritch School of Medicine, Loyola University Chicago, Maywood, Illinois, USA.; 5 Department of Cell and Molecular Pharmacology and; 6 Department of Surgery, Medical University of South Carolina, Charleston, South Carolina, USA.; 7 Cancer Center, University of Colorado Anschutz Medical Campus, Aurora, Colorado, USA.; 8 Department of Medicine, Medical College of Wisconsin, Milwaukee, Wisconsin, USA.

**Keywords:** Cell biology, Immunology, Cellular immune response, Immunotherapy, T cells

## Abstract

The PIM kinase family is critically involved in tumorigenesis, yet its role in primary T cells is understudied. We reported that PIM2, distinct from the other 2 isoforms, inhibits T cell responses to alloantigen. Here, we further established PIM2 as a key negative regulator in antitumor immunity. *Pim2* deficiency in tumor antigen–specific or polyclonal T cells enhanced their ability to control tumor growth in murine breast cancer, melanoma, and leukemia models. *Pim2* deficiency enhanced cytokine production and metabolic activities in tumor-infiltrating CD8 T cells. *Pim2* deficiency increased TCF1 expression and memory-like phenotype in CD8 T cells from lymphoid organs. Mechanistically, PIM2 facilitated LC3 lipidation, P62 degradation, and autophagic flux in T cells, leading to impaired glycolysis and effector cytokine production. Furthermore, through modulating VPRBP kinase phosphorylation, PIM2 inhibited histone methyltransferase activity of EZH2 in CD8 T cells, causing disrupted memory-like phenotype. Notably, the PIM2 inhibitor JP11646 markedly enhanced antitumor T cell response. The immunosuppressive role of PIM2 was validated in human T cells, where inhibition of PIM2 enhanced antitumor responses in engineered human T cells, including melanoma-specific TCR T cells and CD19 CAR T cells. Collectively, PIM2 represents a promising target for improving cancer immunotherapy through enhancing effector differentiation and persistence of CD8 T cells.

## Abstract 2

We identified Pim2 as a key negative regulator of CD8 T-cell antitumor immunity and validated it as a potential therapeutic target for enhancing cancer immunotherapy.

## Graphical abstract

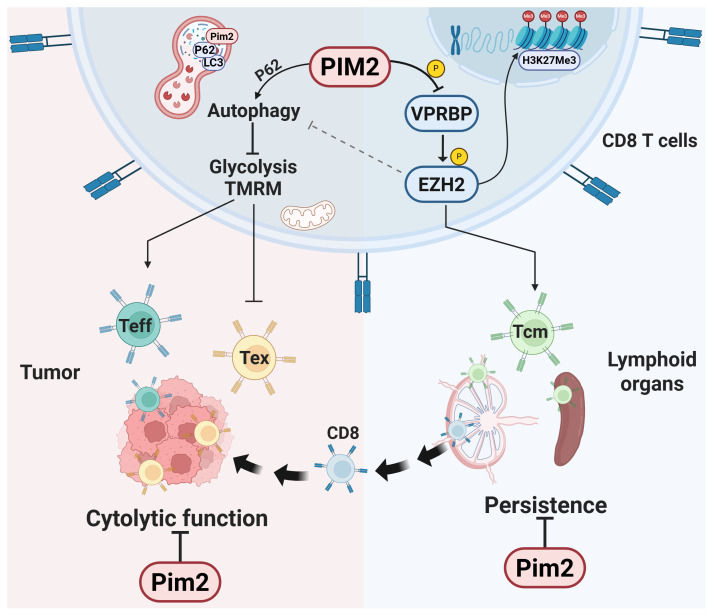

## Introduction

Adoptive cell therapies (ACTs), including therapy with CAR T cells, T cell receptor (TCR) gene–modified T cells, and tumor-infiltrating lymphocytes (TILs), represent promising strategies to orchestrate the antitumor immune response. However, chronic antigen stimulation and stress conditions in the tumor microenvironment (TME), including nutrient deprivation, hypoxia, acidified environment, etc., promote T cell differentiation into short-lived, terminally exhausted T cells (1). Progenitor exhausted (Tpex) (2), stem-like (3), and memory-like T cells (4, 5) are critical for successful ACT through replenishing antitumor effector T cells and sustaining CD8 T cell responses. The development of memory-like or Tpex cells typically happens in the lymphoid organs with the secession of antigen stimulation and supportive cytokines. Effort is still needed to further understand the molecular mechanism regulating CD8 T cell differentiation in tumor and lymphoid organs and to develop therapeutic strategy to simultaneously enhance effector and memory T cell differentiation after ACT.

The Provirus Integration sites for Moloney murine leukemia virus (PIM) kinases are a highly conserved family of serine/threonine kinases (6, 7). The PIM kinase family is composed of 3 isoforms, PIM1, PIM2, and PIM3, which share high homology at the amino acid sequence level (8) and have functional redundancy in promoting tumor cell survival and growth (9). We previously reported key roles of PIM kinases in modulating antitumor immune response, in that deficiency of *Pim1/2/3* improved CD8 T cell stemness and efficacy in ACT (10). Given toxicity and limited efficacy of pan-PIM inhibitors in clinical trials for cancer treatment, targeting a specific PIM isoform could provide a safer and more effective treatment approach. In studying individual Pim isoforms, we found that PIM2 isoform negatively regulates T cell responses, in contrast to 2 other PIM isoforms, following allogeneic hematopoietic cell transplantation (allo-HCT) (11). PIM2 is highly involved in the survival and proliferation of different types of cancer cells, such as B cell lymphoma (12), multiple myeloma (13), and prostate cancer (14), suggesting that it could be an interesting therapeutic target for cancer therapy (15).

However, the role of PIM2 in primary T cells remains largely undefined. PIM2 together with PIM1 in murine T cells (16) and PIM2 in human Tregs (17) provide a survival signal in response to rapamycin-sensitized cell death. PIM2 phosphorylates and stabilizes the regulator of suppressor of cytokine signaling 1 in T cells (18); PIM2 as a downstream target of miRNA-26b-5p restricts T cell immunity against hepatocellular carcinoma (19). These observations suggest PIM2 may play an inhibitory role in T cell response. However, PIM2 also negatively impacts Treg stability and function (20), and PIM2 together with PIM1 promotes cytotoxic T lymphocyte molecular expression in activated T cells (21), suggesting that PIM2 may positively regulate T cell response. Due to the importance of PIM2 in T cell biology and controversial data, it is critical to elucidate the specific role of PIM2 in CD8 T cell immunity and explore pharmacological strategies to selectively target the PIM2 isoform in cancer immunotherapy.

In this study, we identified PIM2 as a modulator of CD8 T cell antitumor immunity and a potential therapeutic target to enhance cancer immunotherapy. Mechanistically, *Pim2* deficiency was associated with augmented CD8 T cell effector differentiation within the TME and increased memory-associated features in lymphoid organs. Our data suggest that PIM2 promotes autophagic flux, a stress-induced process that may limit glycolytic metabolism and effector differentiation. Additionally, PIM2 interacted with VPRBP/EZH2, potentially impairing EZH2 activity and memory T cell formation. Pharmacologic or genetic inhibition of PIM2 enhanced antitumor immunity in both murine and human CD8 T cells. Collectively, these findings establish PIM2 as a promising target for improving cancer immunotherapy by enhancing effector differentiation and persistence of CD8 T cells.

## Results

### PIM2 suppressed T cell function in controlling tumor growth.

**Figure 1:**
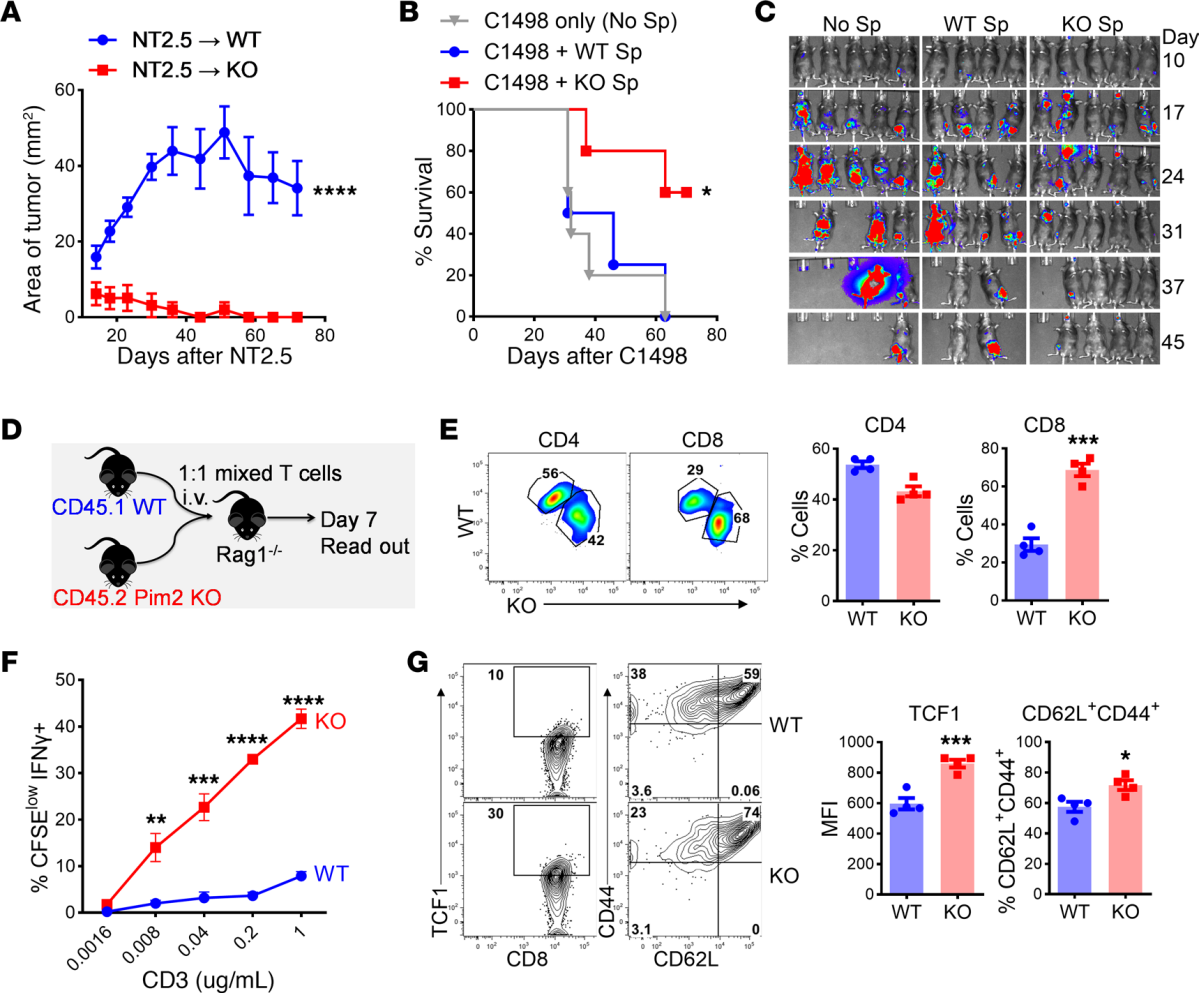
PIM2 negatively regulates T cell antitumor responses. (**A**) WT and *Pim2*-KO FVB mice were infused with NT2.5 mammary cancer cells under the fourth mammary fat pads. Tumor growth was monitored. Data represent 2 independent experiments with *n* = 10 for each group. (**B** and **C**) B6 mice were lethally irradiated and i.v. injected with C1498^luc^ leukemic cells and BM cells from WT B6 mice with or without CD25-depleted splenocytes from WT or *Pim2*-KO B6 mice. Recipient survival (**B**) and leukemia growth (**C**) were monitored through BLI. Data represent 2 independent experiments with total *n* = 10 for each group. (**D**) CD45.1^+^ WT and CD45.2^+^ *Pim2*-KO T cells from B6 mice were mixed 1:1 and transferred into Rag1^–/–^ B6 mice. (**E**) Percentages of WT and *Pim2*-KO cells within gated CD4^+^ and CD8^+^ T cells in spleens on day 14 are shown. Data represent 2 independent experiments with *n* = 8. (**F**) CFSE-labeled splenocytes from WT and *Pim2*-KO mice were stimulated with anti-CD3 for 72 hours, and percentages of CFSE^lo^IFN-γ^+^ cells in gated CD8 T cells are shown. (**G**) Expression of TCF1 and CD44/CD62L is shown in gated CD8 T cells with 1 μg/mL anti-CD3 stimulation. Data represent 2 independent experiments (**E**–**G**). Data were analyzed by 2-tailed Student’s *t* test (**E** and **G**), 2-way ANOVA (**A** and **F**), and log-rank test for survival curves (**B**). Data are shown as mean ± SEM from biological replicates. *P < 0.05, **P < 0.01, ***P < 0.001, ****P < 0.0001.

We previously reported that PIM2 potently suppresses T cell response in causing graft versus host disease (GVHD) after allo-HCT (11). We hypothesized that *Pim2* deficiency would enhance antitumor T cell response. NT2.5, a HER-2/neu-expressing mammary carcinoma cell line on FVB background (22), was orthotopically injected into the mammary fat pad of FVB WT and *Pim2*-KO mice. We have shown that *Pim2* deficiency does not impact T cell development or hemostasis in unmanipulated mice (11). However, in contrast to WT mice, the *Pim2*-KO mice exhibited significantly smaller tumors and eventually rejected tumors (Figure 1A). To validate the function of PIM2 in negatively regulating T cell antitumor response, we utilized a murine autologous HCT model to examine how *Pim2* deficiency in the donor graft impacted graft versus leukemia response. Splenocytes from B6 WT or *Pim2*-KO mice were transferred as cellular immunotherapy into syngeneic recipients that were i.v. injected with acute myeloid leukemia cells (C1498^Luc^, H2K^b+^). Consistently, transfer of *Pim2*-KO splenocytes significantly improved leukemia control compared with WT splenocytes, as indicated by lower bioluminescence imaging (BLI) signaling and increased survival in these recipients (Figure 1, B and C).

To directly examine how PIM2 intrinsically impacts T cell activity in vivo, we mixed purified WT (CD45.1^+^) and *Pim2*-KO (CD45.2^+^) T cells at a 1:1 ratio and transferred them into syngeneic *Rag1*-KO mice (Figure 1D). Fourteen days later, we analyzed T cell populations in recipient spleen and found that frequencies of *Pim2*-KO cells were significantly more abundant than WT controls within CD8^+^ but not CD4^+^ T cells (Figure 1E), indicating an intrinsic role of PIM2 in restricting CD8 T cell response. Because CD8 T cells play a central role in antitumor immunity, we focused our study on illustrating the mechanistic action of PIM2 in suppressing CD8 T cell response. We observed that *Pim2* deficiency substantially enhanced CD8 T cell activation upon anti-CD3/CD28 stimulation in vitro, as reflected by increased proliferation and cytokine production (Figure 1F). Under the culture condition, *Pim2*-KO CD8 T cells maintained higher levels of CD62L and TCF1 expression compared with WT controls (Figure 1G), indicating a preserved progenitor-like feature in *Pim2*-KO CD8 T cells upon activation (23).

### PIM2 restrained antigen-specific CD8 T cell response.

**Figure 2:**
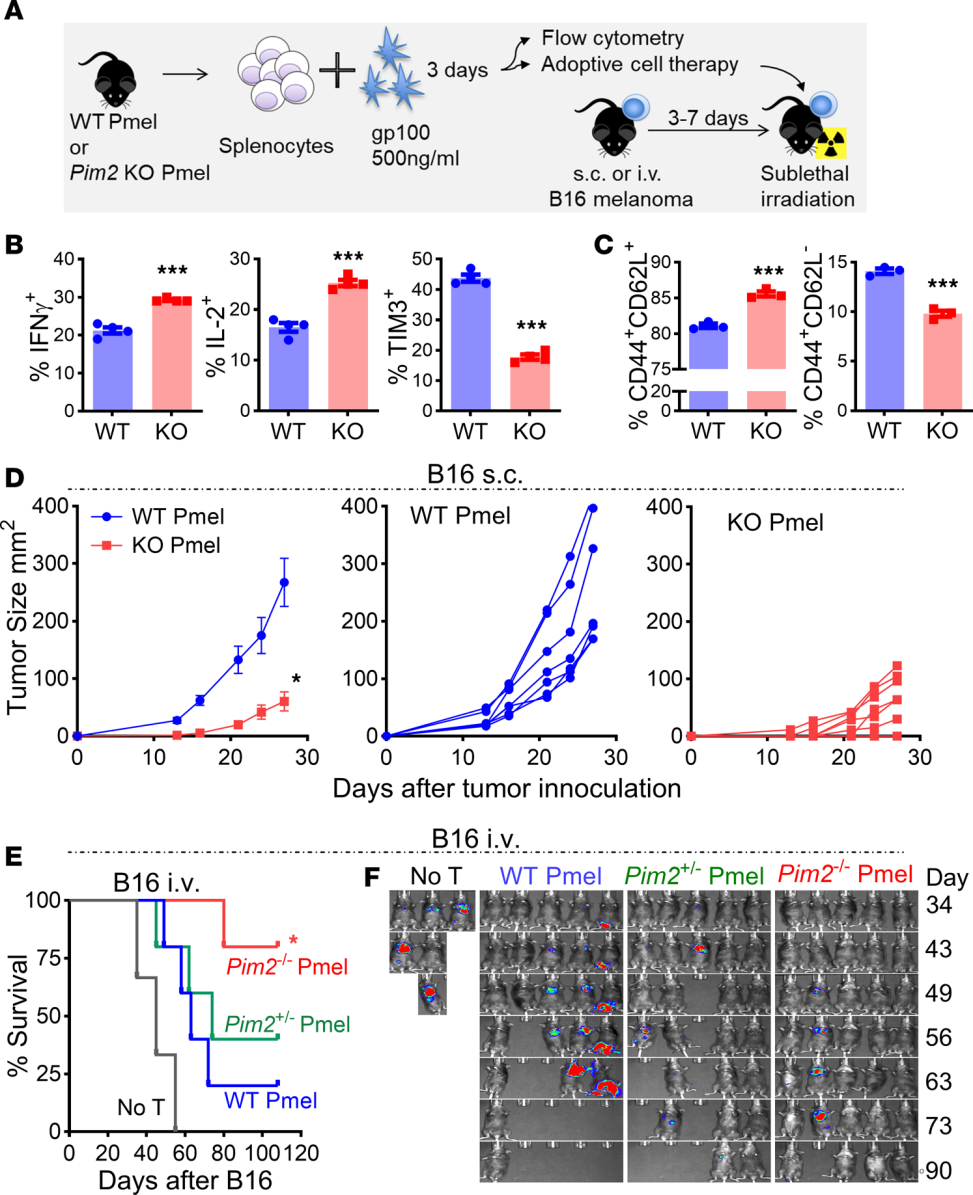
*Pim2* deficiency enhances melanoma control by tumor antigen–specific CD8 T cells after ACT. (**A**) Ly5.1 B6 mice were s.c. infused with a B16F10 tumor on the flank, followed by sublethal irradiation at 600 cGy and adoptive transfer of gp100 peptide preactivated WT or *Pim2*-KO CD8^+^ Pmel T cells on day 7. (**B** and **C**) The phenotypes of CD8^+^ Pmel cells after gp100 peptide stimulation were evaluated by flow cytometry. Data represent 3 independent experiments. (**D**) Tumor growth was monitored. Data represent 3 independent experiments with WT *n* = 18 and KO *n* = 18. (**E** and **F**) Ly5.1 B6 mice were i.v. injected with B16F10, followed by sublethal irradiation at 600 cGy and adoptive transfer of gp100 peptide preactivated WT, *Pim2*^+/–^, or *Pim2*^–/–^ CD8^+^ T cells on day 3. Survival (**E**) and melanoma growth (**F**) in mice were monitored via BLI. Data represent 2 independent experiments with WT *n* = 10, *Pim2*^+/–^ *n* = 5, and *Pim2*^–/–^ *n* = 10. Data were analyzed by 2-tailed Student’s *t* test (**B** and **C**), 2-way ANOVA (**D**), and log-rank test for survival curves (**E**). Data are shown as mean ± SEM from biological replicates. *P < 0.05, ***P < 0.001.

To further investigate how PIM2 regulates antitumor response of CD8 T cells, we generated *Pim2*-KO Pmel TCR Tg mice. Upon gp100 stimulation in vitro (Figure 2A), *Pim2*-KO CD8 T cells secreted more IFN-γ and IL-2 cytokines and expressed less exhaustion marker TIM3 compared with WT controls (Figure 2B). *Pim2*-KO CD8 T cells also maintained higher CD62L expression than WT cells after gp100 stimulation (Figure 2C). We then examined whether *Pim2* deficiency would improve antitumor function of CD8 T cells. The B16F10 melanoma cells were s.c. injected into the flank of Ly5.1 B6 mice. Seven days later, these mice received sublethal irradiation followed by transfer of activated WT or *Pim2*-KO CD8 T cells (Figure 2A). Compared with WT controls, *Pim2*-KO CD8 T cells were markedly more effective in controlling melanoma growth (Figure 2D). By injecting B16F10 cells intravenously, we were able to examine the potency of *Pim2*^–/–^ CD8 T cells in controlling the growth of aggressive melanoma that mimics metastatic tumors in lungs. Consistently, we observed significantly improved antitumor function of *Pim2*-KO (*Pim2*^–/–^) CD8 T cells, as reflected by reduced tumor signaling monitored by BLI and improved survival of these tumor-bearing mice (Figure 2, E and F). In contrast, CD8 T cells with Pim2 heterozygous deficiency (*Pim2*^+/–^) showed comparable antitumor ability to WT controls, suggesting that complete loss of *Pim2* is required to enhance antitumor response of CD8 T cells (Figure 2, E and F).

To verify whether PIM2 indeed plays a negative role in antitumor immunity of CD8 T cells, we generated a *Pim2*-knockin (*Pim2*-KI) mouse model in which PIM2 is overexpressed specifically in T cells (Supplemental Figure 1, A and B; supplemental material available online with this article; https://doi.org/10.1172/JCI192928DS1). We found that PIM2 overexpression attenuated the activation of Pmel T cells following gp100 stimulation, as shown by lower IFN-γ and TNF-α secretion than WT controls (Supplemental Figure 2, A and B). Furthermore, *Pim2*-KI CD8 T cells contained fewer CD62L-expressing subsets, including naive and central memory-like cells (Supplemental Figure 2C). Consistently, adoptive transfer of *Pim2*-KI CD8 T cells was less effective than that of their WT counterparts for controlling B16F10 melanoma growth established either s.c. or i.v. (Supplemental Figure 2, D and E). Taken together, these data suggest that PIM2 negatively regulates activation and CD62L expression of CD8 T cells in vitro and PIM2 restrains activity of antigen-specific CD8 T cells in controlling tumor growth in vivo.

### PIM2 suppressed effector cytokine production by CD8 T cells in the TME after ACT.

**Figure 3:**
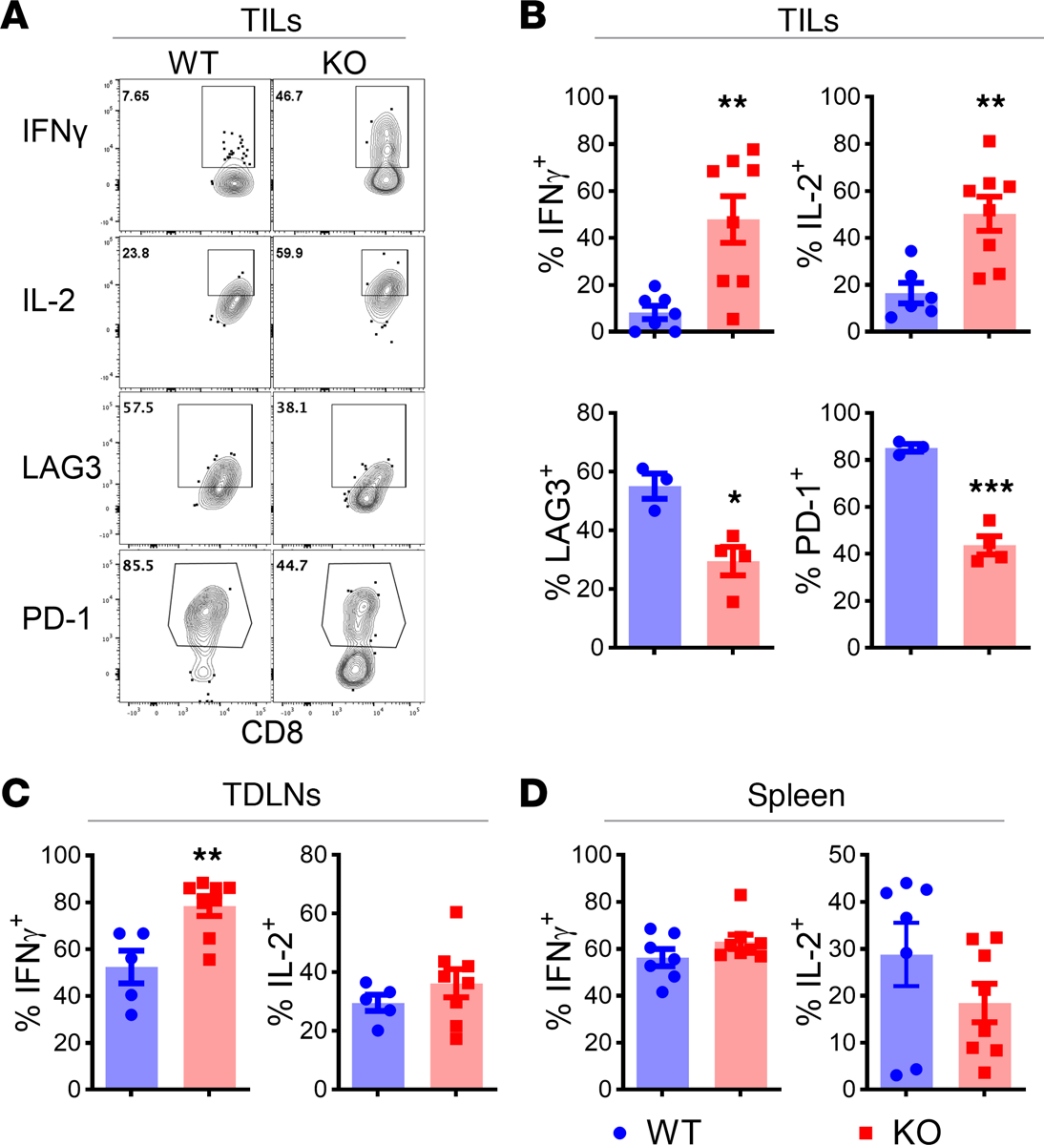
*Pim2* deficiency increases effector cytokines but decreases exhaustion features in CD8^+^ TILs. Ly5.1 B6 mice were s.c. infused with a B16F10 tumor on the flank, followed with sublethal irradiation at 600 cGy and adoptive transfer of gp100 peptide preactivated WT or *Pim2*-KO CD8^+^ Pmel T cells on day 7. At day 21 after ACT, tumors, TDLNs, and spleens were isolated from tumor-bearing mice for flow cytometry analysis. (**A** and **B**) Representative flow figures and bar graphs showing IFN-γ, IL-2, LAG3, and PD-1 expression in gated live Ly5.2^+^ CD8 T cells in tumors. (**C** and **D**). IFN-γ and IL-2 expression in gated live Ly5.2^+^ CD8 T cells in TDLNs and spleens. Data represent 2 independent experiments with WT *n* = 7 and KO *n* = 8. Data were analyzed by 2-tailed Student’s *t* test and are shown as mean ± SEM from biological replicates. *P < 0.05, **P < 0.01, ***P < 0.001.

To elucidate the underlying mechanisms by which PIM2 negatively impacts antitumor immunity of CD8 T cells, we examined how PIM2 impacts CD8 T cell differentiation in the TME. B16F10 was s.c. injected into the flank of Ly5.1^+^ B6 mice. Seven days later, activated WT or *Pim2*-KO Pmel cells were i.v. injected into these tumor-bearing mice after sublethal irradiation. Three weeks after ACT, we found that *Pim2*-KO Pmel cells infiltrated into the tumor secreted more effector cytokines IFN-γ and growth factor IL-2 than WT controls (Figure 3, A and B). *Pim2* deficiency also increased Pmel T cell production of IFN-γ in the tumor-draining lymph nodes (TDLNs) but not in the spleen, suggesting that an antigen encounter is required for the differentiation of these CD8 T cells toward effector cells (Figure 3, C and D). In the TME, *Pim2*-KO Pmel T cells expressed lower levels of exhaustion-associated markers including LAG3 and PD-1 compared with WT controls (Figure 3, A and B). We isolated total CD8 T cells from TDLNs and spleens of the tumor-bearing mice for RNA-seq (Supplemental Figure 3). CD8 T cells had elevated expression of genes associated with T cell activation, including *Jun*, *Gzmb*, and *Cd74* in the TDLNs, and *Cd69*, *Cd28*, *Fos*, and *Fosb* in the spleens of the recipients transferred with *Pim2*-KO Pmel T cells (Supplemental Figure 3, A and C). Pathway enrichment analysis (24) indicated that CD8 T cells from the recipients of *Pim2*-KO Pmel cells had significant upregulation of genes associated with aerobic respiration, respiratory electron transport, positive regulation of leukocyte activation and differentiation, TCR signaling, and cell cycle (Supplemental Figure 3, B and D). We suggest that the transfer of a small number of *Pim2*-KO Pmel cells was sufficient to influence the gene transcription of the entire CD8 T cell population, including the majority (>95%) of host-derived WT CD8 T cells, thereby enhancing T cell activation and immunity in tumor-bearing mice.

To exclude the model-specific phenomenon and validate our findings, we crossed *Pim2*-KO mice (FVB background) with HER-2/neu transgenic mice in which clone 100 TCR Tg T cells specifically recognize MHC-I–restricted HER-2/neu expressed on a NT2.5 breast cancer cell line (25, 26). After stimulation with HER2/neu-derived peptide (RNEU_420-429_) in vitro, *Pim2*-KO CD8 T cells were more activated than WT controls, as reflected by increased proliferation and IFN-γ production (Supplemental Figure 4, A and B). Adoptive transfer of *Pim2*-KO CD8 clone 100 T cells showed markedly increased ability to control the growth of orthotopically implanted NT2.5 breast cancer than WT CD8 T cells in vivo (Supplemental Figure 4C). In the TME, deficiency of *Pim2* increased CD8 T cell effector differentiation, as shown by increased granzyme B, TNF-α, IFN-γ, and IL-2 cytokines (Supplemental Figure 4, D and E). Taken together, these data reveal that PIM2 constrains effector cytokine production by tumor-infiltrating CD8 T cells.

### PIM2 restrained memory-like phenotype and persistence of CD8 T cells after ACT.

**Figure 4:**
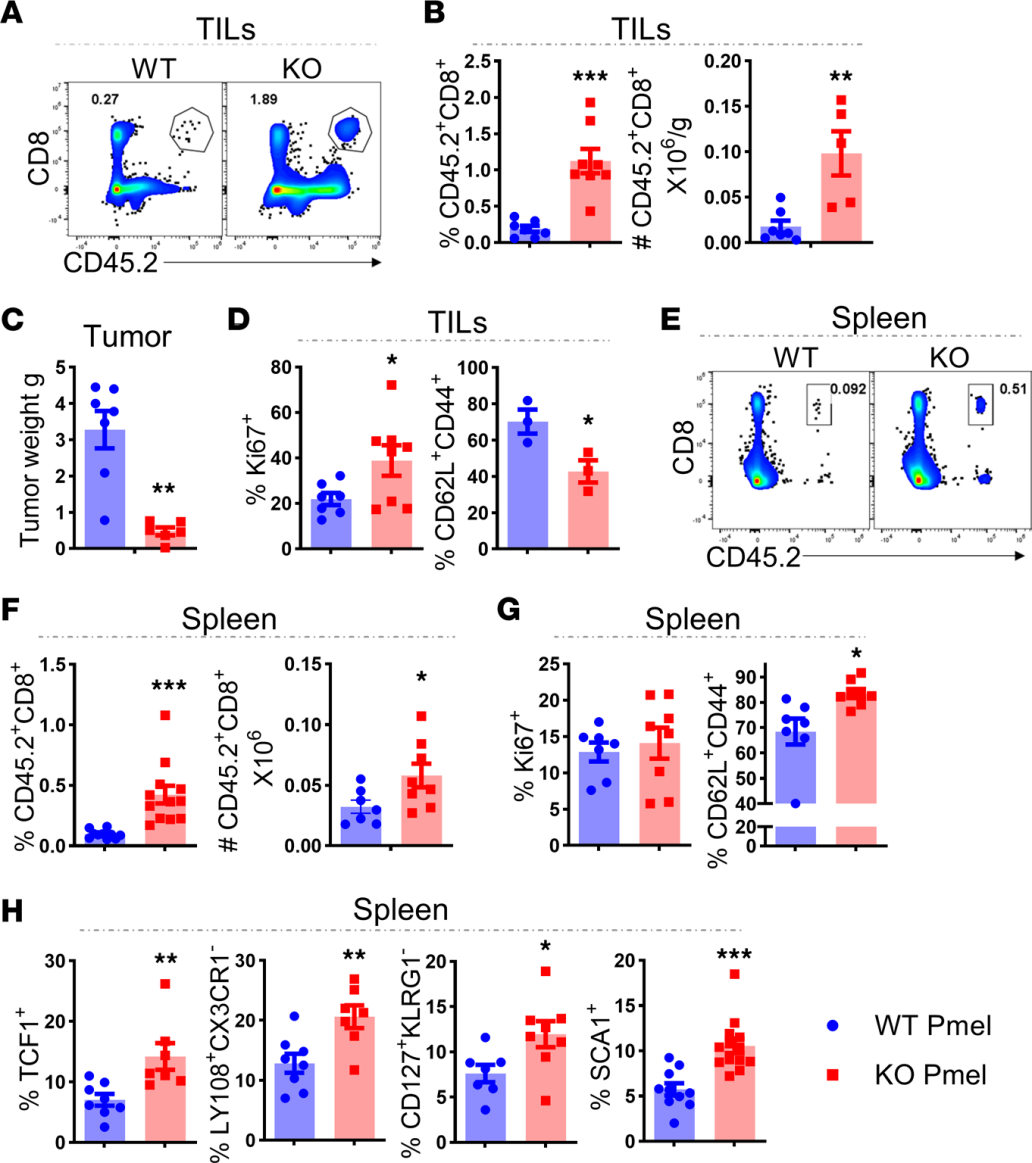
*Pim2* deficiency enhances persistence of CD8 T cells after ACT. The experiments were set up as described for Figure 3. At day 21 after ACT, tumors and spleens were isolated for flow cytometry analysis. (**A** and **B**) Representative flow figures and bar graphs showing frequencies of CD45.2^+^CD8^+^ T cells within gated total live cells and numbers of CD45.2^+^CD8^+^ T cells per gram of tumor. (**C**) Tumor weight in each mouse is shown. (**D**) Frequency of proliferating (Ki67^+^) and memory-like (CD44^+^CD62L^+^) cells in CD45.2^+^CD8^+^ T cells TILs. (**E** and **F**) Representative flow figures and bar graphs showing the frequencies and absolute numbers of CD45.2^+^CD8^+^ T cells within spleens. (**G**) Frequency of proliferating (Ki67) and memory-like (CD44^+^CD62L^+^) cells in CD45.2^+^CD8^+^ T cells are shown in spleens. (**H**) Frequency of TCF-1^+^, LY108^+^CX3CR1^–^, and CD127^+^KLRG1^–^ cells in gated CD45.2^+^CD8^+^ T cells and Sca1^+^ in gated CD62L^+^CD45.2^+^CD8^+^ T cells is shown in spleens. Data represent 2 independent experiments with WT *n* = 7 and KO *n* = 8. Data were analyzed by 2-tailed Student’s *t* test and are shown as mean ± SEM from biological replicates. *P < 0.05, **P < 0.01, ***P < 0.001.

We further examined how PIM2 regulates the persistence of CD8 T cells, a key feature required for successful T cell therapy. WT or *Pim2*-KO Pmel T cells were transferred into B16F10-bearing mice with CD45.1 congenic marker. We observed that tumor-infiltrated antigen-specific T cells, as reflected by frequencies and numbers of CD45.2^+^ CD8 T cells, were significantly increased in the mice transferred with *Pim2*-KO Pmel cells (Figure 4, A and B), which was consistent with smaller tumor size (Figure 4C). Similarly, the frequencies and numbers of *Pim2*-KO CD8 T cells were also increased in the recipient spleens (Figure 4, E and F). Subsequently, we examined whether PIM2 limits the expansion of T cells and found that *Pim2*-KO CD8 T cells in tumors, but not in spleens, had increased expansion, as shown by higher Ki67 expression (Figure 4, D and G). Consistently, in mice with B16 i.v. infusion, transferred *Pim2*-KO Pmel cells persisted better than WT controls, as indicated by increased frequencies of Tg CD8 T cells in both peripheral blood and spleen (Supplemental Figure 5, A–C), and the *Pim2*-KO Pmel cells expressed lower levels of exhaustion-associated markers, including PD-1 and LAG3, than WT controls (Supplemental Figure 5D).

Since memory T cells have self-renewing potential and can continuously differentiate into effector T cells, we examined the central memory-like or Tcm features (CD44^+^CD62L^+^) of these Pmel CD8 T cells after ACT. Consistent with increased persistence, *Pim2*-KO CD8 T cells exhibited elevated memory-like features compared with WT controls in the spleen, one of the primary locations for the memory T cells (Figure 4G). However, in tumors, these central memory-like features were significantly downregulated in the *Pim2*-KO cells compared with the WT CD8 T cells, suggesting more effector differentiation from the KO T cells when encountering tumor antigens (Figure 4D). Consistent with increased memory-like features, *Pim2*-KO CD8 T cells also expressed increased levels of TCF1 (27), LY108 (28), CD127 (29) and SCA-1 (30), markers associated with progenitor-like T cells in spleens, but not in tumors (data not shown), of B16-bearing mice following ACT (Figure 4H). Taken together, these data suggest that PIM2 negatively affects the persistence of tumor antigen–specific T cells potentially by influencing memory- or progenitor-like T cell differentiation in lymphoid organs.

### PIM2 attenuated metabolic adaption of CD8 T cells in response to tumor antigen.

To understand the molecular mechanism underlying how PIM2 suppresses CD8 T cell immunity, we performed proteomics analysis and studied the differentially expressed proteins between the *PIM2*-deficient and -competent Pmel cells after gp100 activation. Compared with WT controls, *Pim2*-KO CD8 T cells had higher expression of proteins related to activation and effector, such as JUNB, CD99, TAF7, and GZMB, and proteins related to mitochondrial complex I biogenesis, such as NDUFB8, NDUFA8, and NDUFAF4 (Supplemental Figure 6A). In addition, *Pim2*-KO CD8 T cells had decreased expression of proteins related to T cell exhaustion, such as TOX (31) and IKZF2 (32) (Supplemental Figure 6A). Pathway enrichment analysis revealed that proteins increasingly expressed in *Pim2*-KO CD8 T cells were associated with immune activation, translational initiation, and negative regulation of apoptosis, while proteins increasingly expressed in WT CD8 T cells were associated with PTEN regulation, ER stress, and cellular response to hypoxia (Supplemental Figure 6, B and C). Importantly, *Pim2* deficiency influenced CD8 T cell metabolism, including upregulating proteins involved in glycolysis, amide metabolism, and cellular catabolism and downregulating proteins involved in cholesterol biosynthesis and lipid metabolism (Supplemental Figure 6, B and C).

**Figure 5:**
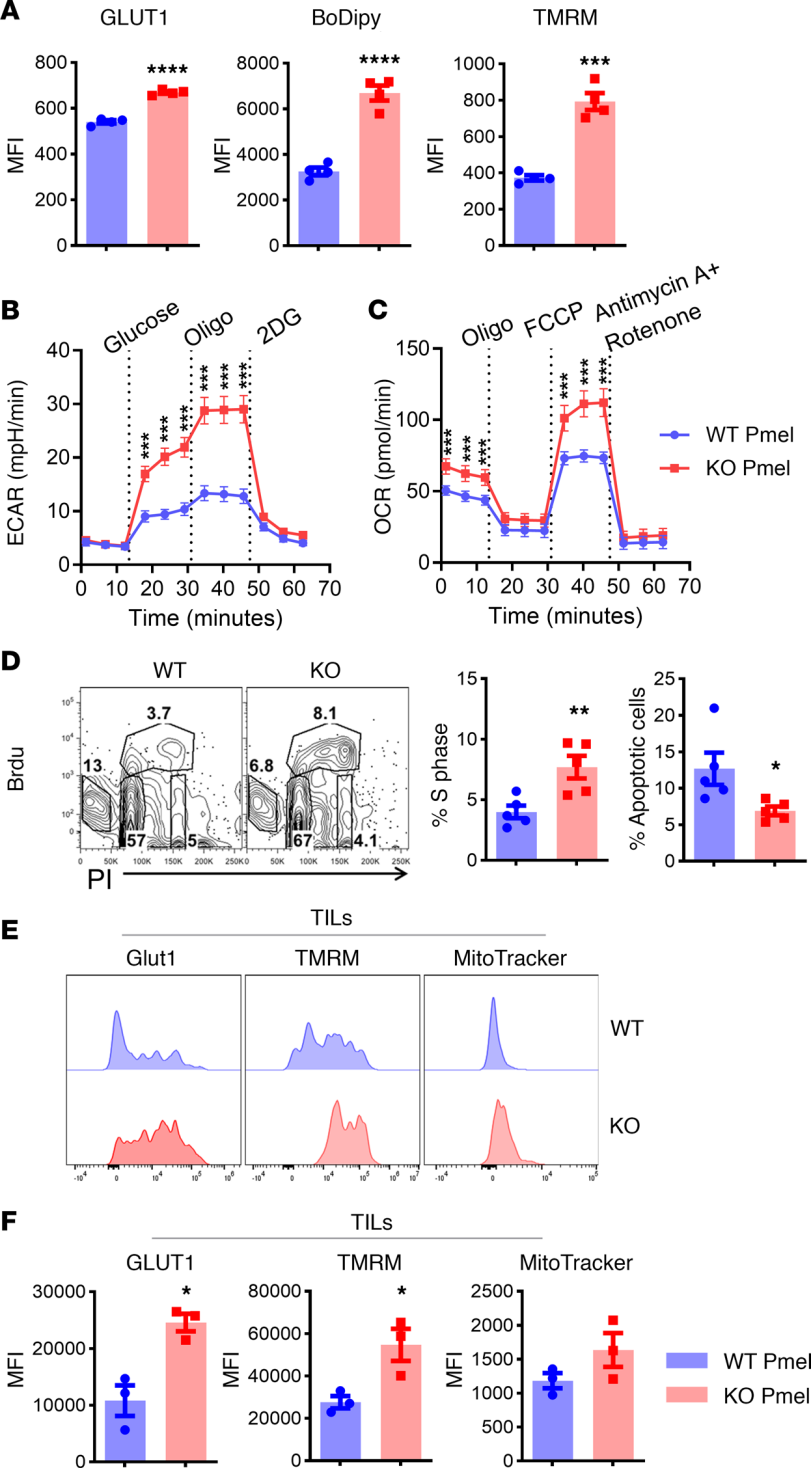
*Pim2* deficiency increases metabolic activities of CD8 T cells after tumor antigen stimulation. (**A**–**C**) Splenocytes from WT or *Pim2*-KO Pmel mice were activated with 500 ng/mL gp100 peptide for 3 days. These activated CD8 T cells were subjected to flow staining for Glut1, BODIPY, and TMRM (**A**) and a Seahorse assay for measuring ECAR and OCR (**B** and **C**). In addition, on day 3, cell culture was pulsed with BrdU, and cell cycle progression was examined. Oligo, oligomycin; 2DG, 2-deoxy-d-glucose; FCCP, carbonyl cyanide 4-(trifluoromethoxy)phenylhydrazone. (**D**) Representative flow figures and bar graphs showing the frequency of cells in S phase and apoptosis. Data represent 2 independent experiments (**A**–**D**). (**E** and **F**) Ly5.1 B6 mice were s.c. infused with B16F10 tumors on the flank, followed by sublethal irradiation at 600 cGy and adoptive transfer of gp100 peptide preactivated WT or *Pim2*-KO pmel CD8 T cells on day 7. On day 21 after ACT, expression of Glut1, TMRM, and MitoTracker was observed in gated CD45.2^+^CD8^+^ T cells in tumors. Data represent 2 independent experiments with WT *n* = 7 and KO *n* = 8. Data were analyzed by 2-tailed Student’s *t* test (**A**, **D**, and **F**) and 2-way ANOVA (**B** and **C**). Data are shown as mean ± SEM from biological replicates. *P < 0.05, **P < 0.01, ***P < 0.001, ****P < 0.0001.

Metabolic reprogramming is critically required to meet the increased demands for bioenergy and biochemicals during T cell activation. Cellular metabolites also influence the transcriptional and epigenetic programs for differentiation and effector function of CD8 T cells during antitumor response (33, 34). To validate our proteomics data showing that PIM2 influences CD8 T cell metabolism, we examined metabolic activities of *Pim2*-deficient and -competent Pmel cells after gp100 activation using flow cytometry and Seahorse assay. In line with increased glycolysis-related proteins (Supplemental Figure 6B), *Pim2*-KO CD8 T cells expressed higher levels of the glucose transporter GLUT1 than WT cells (Figure 5A). Consistent with increased activation and effector cytokine production, the *Pim2*-KO CD8 T cells also contained increased neutral lipid and mitochondria activity, as reflected by BODIPY and TMRM staining, respectively (Figure 5A). Compared with WT controls, *Pim2*-KO CD8 T cells had higher levels of ECAR and OCR, indicating increased metabolic activity, including glycolysis and OXPHOS (Figure 5, B and C). Since metabolic reprogramming is crucial for supporting the proliferation and survival during T cell activation, we then examined cell cycle progression in CD8 T cells after gp100 stimulation. Importantly, *Pim2*-KO CD8 T cells had increased S phase progression and reduced apoptotic cells (Figure 5D). Furthermore, following ACT, *Pim2*-KO CD8^+^ TILs exhibited higher glycolysis and mitochondrial activity than WT controls, demonstrated by Glut1 and TMRM expression, respectively, although comparable mitochondrial content, as shown by MitoTracker green staining (Figure 5, E and F). Taken together, we demonstrated that PIM2 may negatively regulate CD8 T cell activation and effector cytokine production through restraining glycolytic and mitochondrial metabolism upon TCR stimulation.

### PIM2 inhibited CD8 T cell effector function through increasing autophagy.

Next, we studied the mechanism underlying elevated metabolic activity and effector differentiation of CD8 T cells in response to *Pim2* deficiency. Previous studies revealed PIM2 as a key promoter of autophagy, in that PIM2 promotes the expression and organization of LC3 and BECLIN-1 and enhances lysosomal acidification in chondrocytes (35) and that it binds and phosphorylates Hexokinase-II on Thr_473 to induce glucose starvation–induced autophagy (36). In CD8 T cells, suppression of autophagy resulted in enhanced antitumor response by shifting CD8 T cells toward glycolytic metabolism and an effector program (37). We therefore hypothesize that CD8 T cells deficient for PIM2 may enhance glycolysis and effector differentiation through inhibiting autophagy. To define the relationship of PIM2 with autophagy within T cells, we evaluated the expression of PIM2 in response to autophagy modulators and found that PIM2 was downregulated in T cells upon activation but was restored when autophagy flux was inhibited by chloroquine (Supplemental Figure 7A). PIM2 expression was not affected by the autophagy inducer metformin (Supplemental Figure 7A).

**Figure 6:**
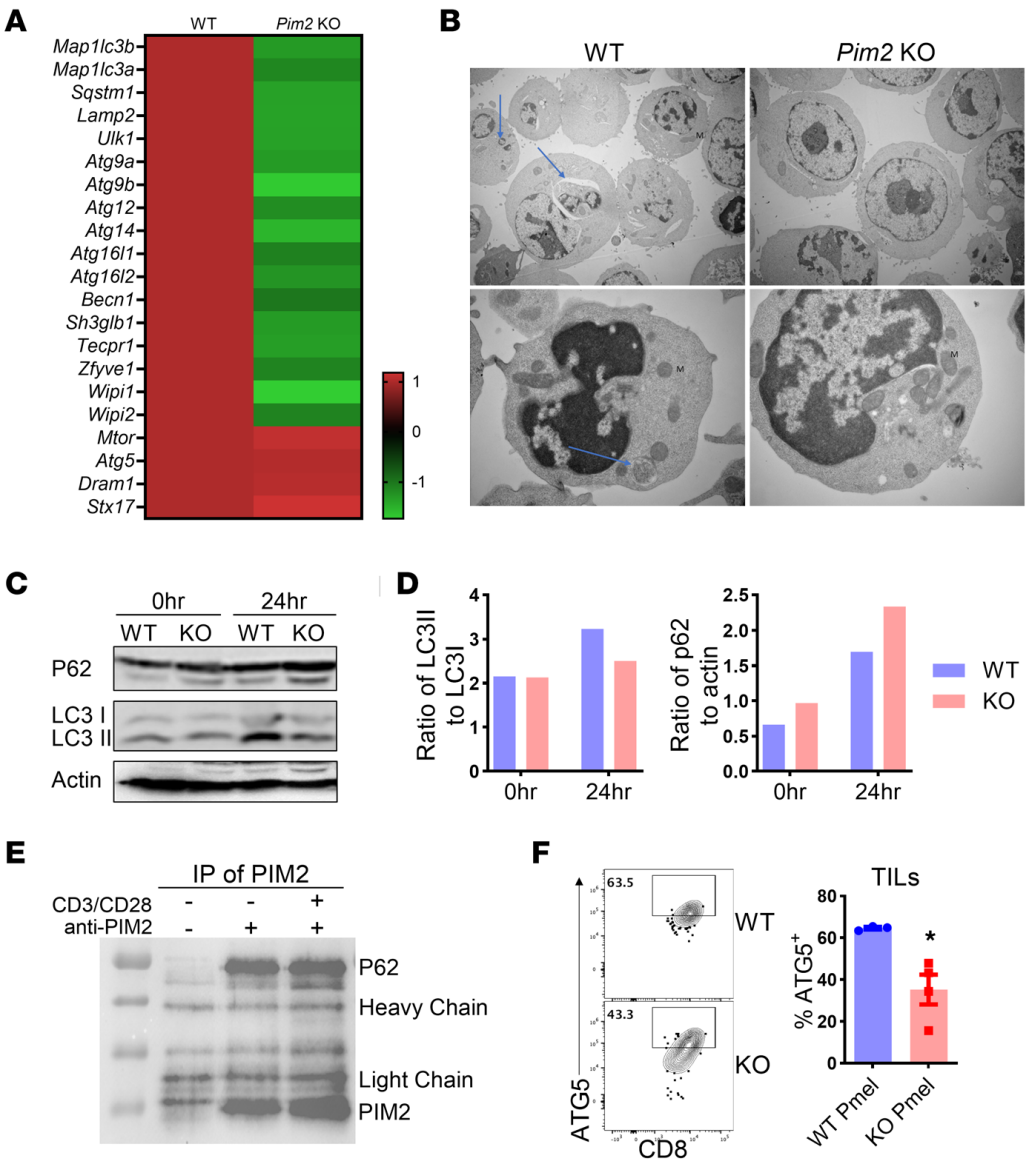
PIM2 negatively regulates autophagic flux in CD8 T cells. (**A** and **B**) T cells from WT or *Pim2*-KO mice were stimulated with anti-CD3/CD28 at 2 μg/mL for 3 days and subjected to bulk RNA-seq and transmission electron microscopy. (**A**) Genes involved in regulating autophagy are displayed in a heatmap. (**B**) Autophagosomes are indicated by arrows. Original magnification, ×5,000 (top row), ×25,000 (bottom row). (**C** and **D**) CD8 T cells from WT or *Pim2*-KO mice were stimulated with anti-CD3/CD28 for 24 hours, and P62 and LC3I/II were examined. (**E**) T cells from *Pim2*-KI mice were stimulated with anti-CD3/CD28 for 24 hours. PIM2 was pulled down, and P62 was examined. Data represent 2 independent experiments. (**F**) Ly5.1 B6 mice were s.c. infused with B16F10, followed by sublethal irradiation and transfer of preactivated Pmel cells on day 7. Frequencies of ATG5^+^ cells in gated CD45.2^+^CD8^+^ TILs are shown on day 21 after ACT. Data represent 2 independent experiments with WT *n* = 7 and KO *n* = 8. Data were analyzed by DESeq2 (**A**) and 2-tailed Student’s *t* test (**F**). Data are shown as mean ± SEM from biological replicates. *P < 0.05.

To further assess the influence of PIM2 on autophagy in T cells, we analyzed bulk RNA-seq data of activated T cells (11) and found lower expression of autophagy-related genes in *Pim2*-KO T cells compared with WT controls, including *Map1lc3b* (encoding microtubule-associated protein light chain 3 beta [LC3b]), *Map1lc3a* (encoding LC3a), *Sqstm1* (encoding P62), *Lamp2* (encoding CD107b), *Ulk1*, and many *Atg* family genes (Figure 6A). Under electron microscopy, autophagosomes were visible in activated WT T cells, but not in *Pim2*-KO cells (Figure 6B). We further examined expression of LC3-I and LC3-II, the lipidated form of LC3-I and a classic autophagosome marker incorporated into the elongating membrane to form autophagosomes (38). The autophagy levels reflected by the ratio of LC3-II (14 kDa) to LC3-I (16 kDa) were decreased in *Pim2*-KO CD8 T cells (Figure 6, C and D) but increased in *Pim2*-overexpressing CD8 T cells (Supplemental Figure 7B) compared with that of WT controls upon activation. We further examined P62, a protein that links ubiquitin protein to autophagosomes and accumulates when autophagy is inhibited (39). Consistently, P62 was more abundant in *Pim2*-KO CD8 T cells (Figure 6, C and D) but less abundant in *Pim2*-KI CD8 T cells (Supplemental Figure 7B) compared with that of WT controls upon activation. Using a coimmunoprecipitation assay, we found that PIM2 formed a complex with P62 in T cells (Figure 6E), suggesting PIM2 may promote autophagy through interacting with P62 in T cells. Consistent with PIM2’s role in inducing T cell autophagy in vitro, *Pim2*-KO CD8 T cells that infiltrated in tumors expressed a significantly lower level of Atg5, a core protein in the autophagy machinery (40), than WT controls (Figure 6F). Recently, amino acid transport was found to acutely repress autophagy in activated CD8 T cells during antigen engagement and inflammatory cytokine stimulation (41). Similarly, we observed that PIM2-deficient CD8 T cells exhibited increased expression of the amino acid transporter CD98 (a heterodimer composed of SLC3A2 and SLC7A5) following antigen stimulation (Supplemental Figure 7C). In addition, *Pim2* deficiency enhanced kynurenine uptake in activated CD8 T cells cultured with IL-2 (Supplemental Figure 7D), suggesting amino acid transport may contribute to the reduced autophagic activity in *Pim2*-KO CD8 T cells.

To validate the contribution of autophagy modulation to PIM2-mediated T cell suppression, we examined whether inducing autophagy in *Pim2*-KO T cells could reverse their enhanced effector function. Through overexpression of Atg5 to induce autophagy (Supplemental Figure 8A), we then observed similar levels of effector cytokines, including IFN-γ and IL-2, produced by *Pim2*-KO versus WT CD8 T cells upon activation in vitro (Supplemental Figure 8B). Consistently, when treated with autophagy inducer, spermidine, the difference between WT and *Pim2*-KO CD8 T cells regarding effector cytokine production was again eliminated (Supplemental Figure 8C). Taken together, our data illuminated that loss of *Pim2* enhances effector cytokine production by T cells at least partially through attenuating autophagy.

### PIM2 suppressed memory-like phenotype on CD8 T cells through modulation of EZH2 activity.

**Figure 7:**
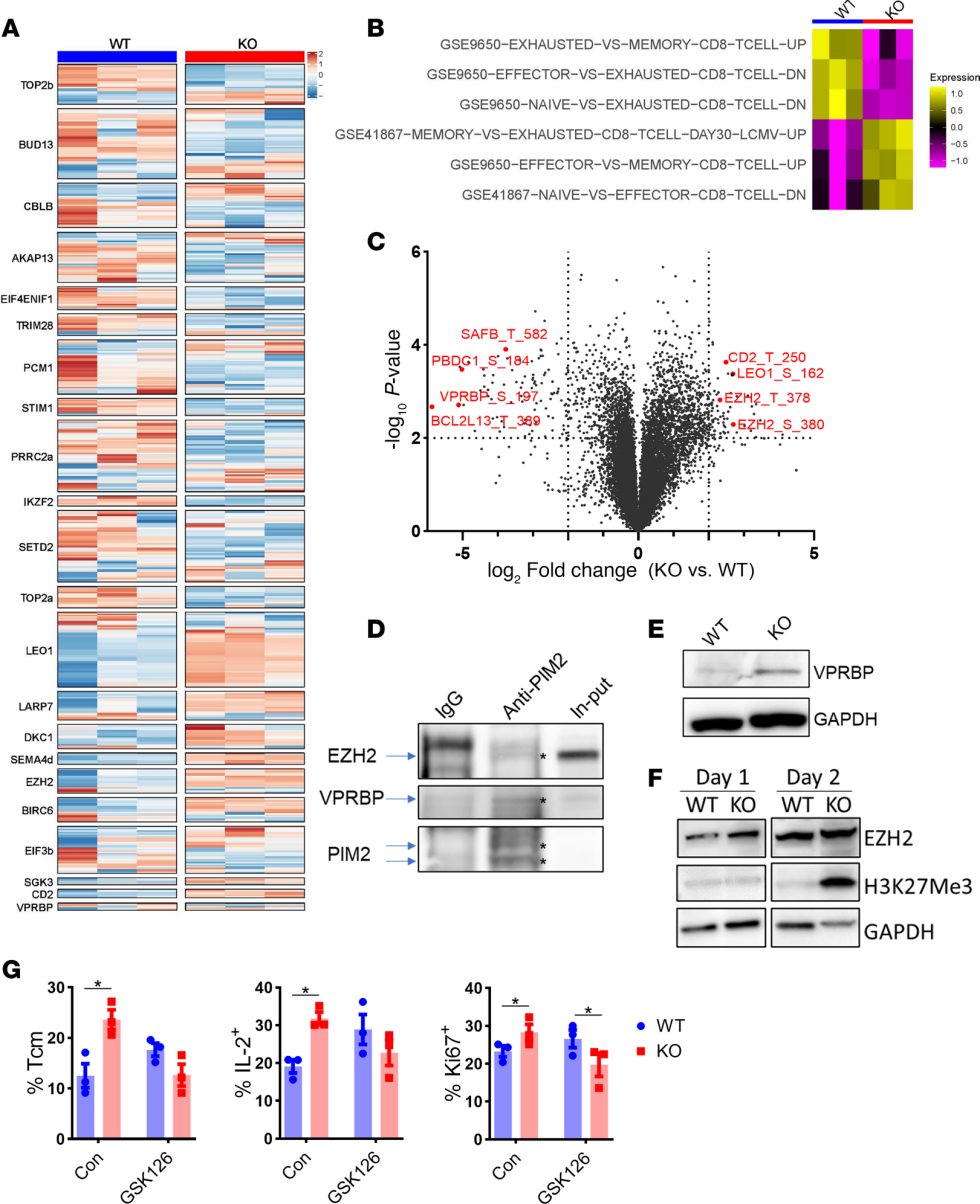
PIM2 negatively regulates VPRBP/EZH2 activity. (**A**–**C**) Splenocytes from WT or *Pim2*-KO Pmel mice were activated with 500 ng/mL gp100 peptide for 3 days and subjected to tandem mass tag–based phosphoproteomics analysis with WT *n* = 5 and KO *n* = 5. (**A**) Differential expression analysis for each phosphosite between WT and KO was conducted, and differentially expressed phosphoproteins reported to regulate T cell response are displayed. (**B**) A pathway analysis was performed in the differentially phosphorylated proteins. The average abundance of the phosphorylated proteins is shown in each pathway. (**C**) All differentially expressed phosphoproteins are shown in a volcano plot. (**D**) WT or *Pim2*-KO CD8 T cells were stimulated with anti-CD3/CD28 for 24 hours. Anti-PIM2 mAb or control IgG was used to pull down PIM2, followed by Western blot detection of EZH2 and VPRBP. The target bands are marked with asterisks. (**E**) Western blot detection of VPRBP was performed in activated WT or *Pim2*-KO Pmel cells at 3 days after gp100 stimulation. (**F**) EZH2 and H3K27Me3 were detected in CD8 T cells after anti-CD3/CD28 stimulation. (**G**) Pmel splenocytes were activated with gp100 peptide plus 5 ng/mL IL-15 with or without 5 μM GSK126 for 5 days. Frequencies of IL-2^+^, IFN-γ^+^, and CD44^+^CD62L^+^ (Tcm) are shown. Data represent 2 independent experiments (**D**–**G**). Data were analyzed unpaired 2-tailed Student’s *t* test (**A** and **G**). Data are shown as mean ± SEM. *P < 0.05.

We observed that *Pim2* deficiency in CD8 T cells not only enhanced their effector function in TME (Figure 3), but also increased memory/progenitor-like phenotype in lymphoid organs after ACT (Figure 4). To further elucidate the molecular mechanisms by which PIM2 influences memory CD8 T cell differentiation, we performed phosphoproteomics analysis and studied the differentially expressed phosphoproteins between the PIM2-deficient and -competent Pmel cells after gp100 activation. Among these, proteins known to regulate T cell responses are displayed in the heatmap (Figure 7A). We found that PIM2 modified phosphorylation of a protein across multiple sites, including both up- and downregulation. *Pim2*-KO CD8 T cells contained mostly lower phosphorylation in proteins reported to be negative regulators of T cells, including CBLB (42), AKAP13 (43), TRIM28 (44), STIM1 (45), and IKZF2 (46), and mostly higher phosphorylation in epigenetic regulators, including LEO1 (47), EZH2 (48), and senescence inhibitory factors, including LAPR7 (49) and DKC1 (50, 51). We then performed pathway analysis and quantified the average abundance level of differentially expressed proteins in each pathway. We found that *Pim2*-KO Pmel cells had substantially increased abundance of phosphoproteins that related to effector and memory cell differentiation but decreased abundance of phosphoproteins related to exhaustion (Figure 7B). Among these differentially phosphorylated proteins, phosphorylation of EZH2, a histone methyltransferase reported to promote CD8 T memory precursor formation (48), was dramatically increased at sites Ser_380 and Thr_378 in *Pim2*-KO Pmel cells than WT controls (Figure 7C). Interestingly, VPRBP, a kinase reported to phosphorylate and stabilize EZH2 (52), had markedly reduced phosphorylation at the Ser_197 site in *Pim2*-KO Pmel cells than WT controls (Figure 7C).

We further hypothesized that PIM2 may interact with VPRBP and EZH2, therefore regulating their phosphorylation and activity in directing CD8 T cell differentiation. To test this, we purified WT CD8 T cells and immunoprecipitated PIM2 24 hours after anti-CD3/CD28 stimulation. Indeed, we found PIM2 interacted with both VPRBP and EZH2 (Figure 7D). In addition to modulating the phosphorylation of VPRBP (Figure 7, A and C), *Pim2* deficiency also led to an increase in total protein levels of VPRBP (Figure 7E). Consistently, *Pim2*-KO Pmel cells had increased expression of EZH2 and its activity, as reflected by H3K27 trimethylation (H3K27me3) (Figure 7F). To explore the possible role of VPRBP in promoting EZH2 activity in CD8 T cells, we inhibited VPRBP kinase activity with B32B3 and found that inhibition of VPRBP reversed the heightened H3K27me3 expression in *Pim2*-KO CD8 T cells (Supplemental Figure 9A). Using an EZH2 inhibitor, GSK126, we further examined how EZH2 inhibition impacts CD8 T cell response. While *Pim2*-KO CD8 T cells had increased Tcm differentiation compared with WT controls during chronic peptide stimulation, addition of GSK126 minimized the difference (Figure 7G). Interestingly, GSK126 also reversed the elevated levels of IL-2 and Ki67 in *Pim2*-KO CD8 T cells (Figure 7G). Consistent with the reported suppressive role of EZH2 in autophagy (53), EZH2 inhibition reversed the heightened P62 accumulation in *Pim2*-KO CD8 T cells and equalized autophagy levels in WT and *Pim2*-KO CD8 T cells (Supplemental Figure 9, B and C). Taken together, our data indicate that increased activity of EZH2 contributes to enhanced memory-like features and effector cytokine production by *Pim2*-KO CD8 T cells.

### Pharmacologic inhibition of PIM2 promoted T cell antitumor immunity.

All the data presented so far were obtained using genetically modified models. To enhance translational relevance, we also pursued pharmacological blockade. JP11646, a non-ATP competitive inhibitor more specific for PIM2 isoform, was shown to inhibit multiple myeloma growth (15). Thus, we evaluated the effect of JP11646 on antigen-specific CD8 T cell response and found that it increased WT T cell proliferation and activation in a dose-dependent manner, but the effect was much less profound on *Pim2*-KO T cells (Supplemental Figure 10). We suggest that JP11646 specifically inhibited the PIM2 isoform. We then elucidated the impact of PIM2 inhibition on antitumor immunity using murine models of ACT. We found that JP11646 treatment in mice markedly improved antitumor efficacy of WT TCR clone 100 T cells in controlling NT2.5 breast cancer (Supplemental Figure 11, A and B). Furthermore, JP11646 treatment also increased the ability of WT Pmel cells to control B16F10 melanoma growth established by either s.c. or i.v. injection (Supplemental Figure 11, C and D). The improvement of antitumor immunity of CD8 T cells by JP11646 administration was as effective as *Pim2* deficiency in both tumor models (Supplemental Figure 11). Furthermore, JP11646 administration did not further enhance antitumor responses mediated by *Pim2*-KO T cells, suggesting that the efficacy of JP11646 requires PIM2 expression in T cells (Supplemental Figure 11E). Collectively, our data indicated that targeting PIM2 with JP11646 promotes T cell antitumor immunity.

### PIM2 negatively regulated human T cell response.

**Figure 8:**
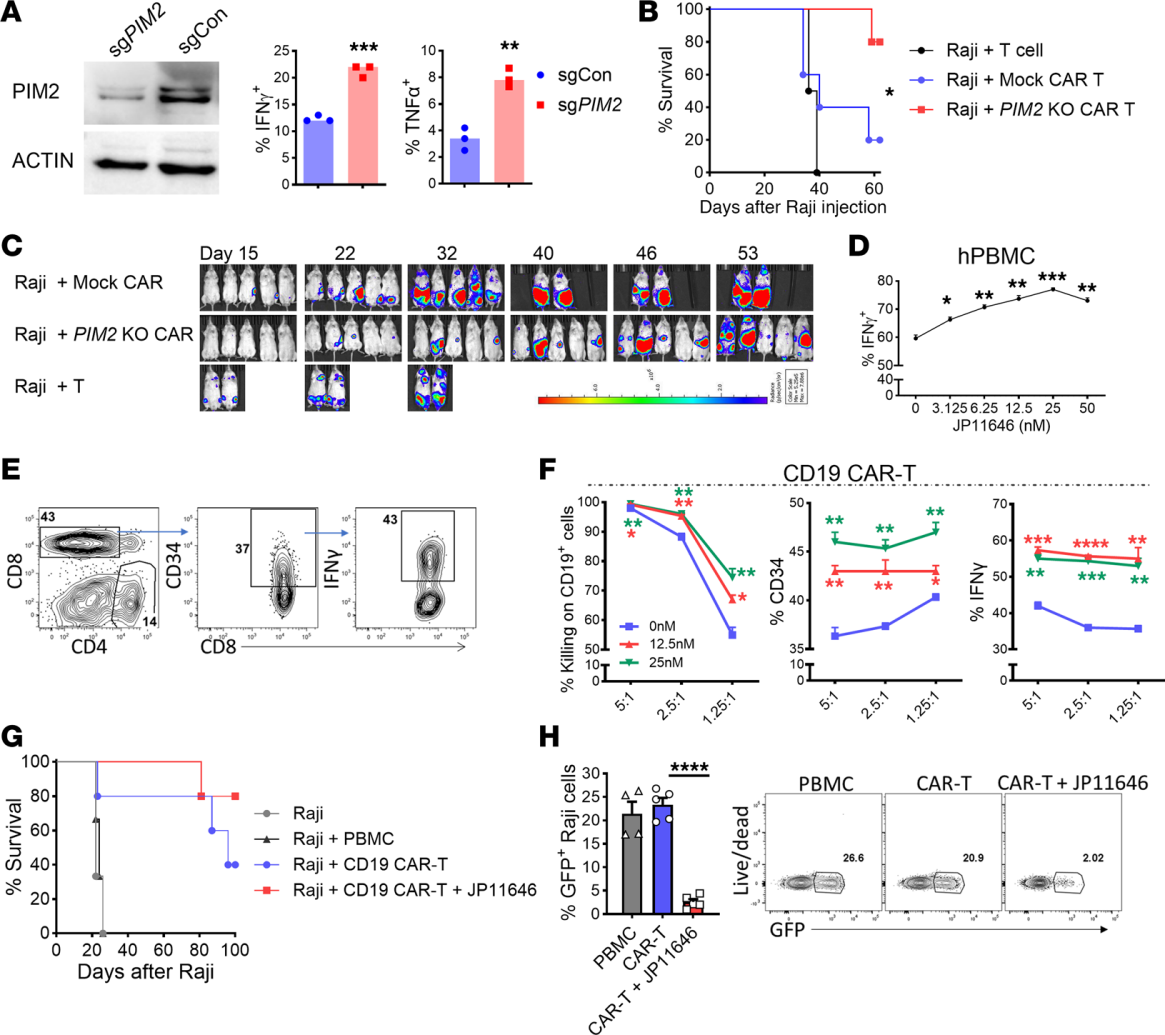
Targeting PIM2 increases human T cell antitumor response. (**A**) Pan T cells were isolated from human PBMCs, transfected with sgRNA to silence PIM2, and stimulated with anti-CD3/CD28 for 3 days. PIM2 expression in T cells and percentages of IFN-γ^+^ and TNF-α^+^ in gated CD8 T cells are shown. (**B** and **C**) Pan T cells were isolated from human PBMCs, transfected with sgRNA to silence *PIM2*, and 48 hours later activated with anti-CD3 beads for 24 hours. Cells were then transduced with control or CD19 CAR vector and expanded in hrIL-7 and hrIL-15 for 7 days. NSG mice were i.v. injected with 0.5 × 10^6^ luciferase-transduced Raji cells, and 6 days later 3 × 10^6^ control T cells or CAR T product (including ~1 × 10^6^ CAR^+^ cells) were infused. Survival and bioluminescence were monitored. (**D**) Human PBMCs were stimulated with 1 μg/mL anti-CD3 for 3 days. Percentages of IFN-γ^+^ cells in gated CD8 T cells are shown. (**E** and **F**) Activated human T cells were transduced with CD19 CAR vector with a truncated CD34 tag and then expanded in IL-2 for 5 days. CAR T cells were cultured with Raji cells at the ratios indicated with or without JP11646 overnight. Gating strategy (**E**) and percentage of killing of CD19^+^ target cells, CD34^+^ cells among CD8^+^ cells, and IFN-γ^+^ cells (**F**) among CD8^+^CD34^+^ cells. Data represent 2 independent experiments (**A**–**F**). (**G**) NSG mice were i.v. injected with 1 × 10^6^ GFP^+^ Raji cells and 3 days later with 1 × 10^6^ CAR^+^ T cells. JP11646 or vehicle was administrated i.p. at 7.5 mg/kg twice a week for 4 weeks. Survival was monitored. (**H**) Percentages of Raji (GFP^+^) cells in peripheral blood are shown on day 28. Data represent 2 independent experiments with *n* = 10 mice/group. Data were analyzed by 2-tailed Student’s *t* test (**A**), 1-way ANOVA (**D** and **H**), 2-way ANOVA (**F**), and log-rank test for survival curves (**B** and **G**). Data are shown as mean ± SEM from biological replicates. *P < 0.05, **P < 0.01, ***P < 0.001, ****P < 0.0001.

To increase translational potential, we further evaluated the role of PIM2 in human CD8 T cell responses. We silenced *PIM2* in human naive T cells using sgRNA and examined their response to anti-CD3/CD28 stimulation (Figure 8A). Loss of *PIM2* markedly increased effector cytokine production in human CD8 T cells (Figure 8A). We then tested how *PIM2* deficiency impacts human CD19 CAR T cells controlling CD19^+^ target cells (Raji) (54). Raji cells were i.v. injected into NSG mice followed by control or PIM2-deficient CD19 CAR T therapy. *Pim2* deficiency markedly reduced Raji tumor burden and improved recipient survival (Figure 8, B and C). Furthermore, inhibition of PIM2 with JP11614 significantly enhanced human CD8 T cell activation in response to anti-CD3 stimulation, as reflected by increased IFN-γ production in a dose-dependent manner (Figure 8D). We then evaluated how PIM2 inhibition impacted human T cell response to tumor-associated antigens. By transducing with lentivirus encoding the TIL1383I TCR, we generated T cells that specifically recognized human melanoma epitope tyrosinase presented by HLA-A2 (55, 56). After coculture with T2 cells pulsed with tyrosinase peptide, we found that addition of the PIM2 inhibitor JP11646 enhanced the expansion of TIL1383I cells (CD34^+^) and their activation, as shown by elevated IFN-γ, CD107a, and CD25 expression (Supplemental Figure 12). Similarly, inhibition of PIM2 increased expansion, cytokine production, and killing activity of CD19 CAR T cells in vitro (Figure 8, E and F). We further examined whether JP11646 could enhance in vivo treatment efficacy of CD19 CAR-T cells and found that mice treated with JP11646 showed improved survival compared with those with vehicle control (Figure 8G). Consistently, JP11646 treatment markedly reduced tumor burden after CAR T therapy (Figure 8H). Taken together, these results indicate that targeting PIM2 promotes human T cell responses to tumor antigens.

## Discussion

In this study, we established that PIM2 plays a key role in suppressing CD8 T cell immunity. Genetic deficiency or pharmacological inhibition of PIM2 enhanced CD8 T cell response; in contrast, overexpression of PIM2 impaired CD8 T cell ability in controlling tumor growth. The protective CD8 T cell subsets against tumors, including effector T cells in tumors and memory-like cells in lymphoid organs, were increased by PIM2 loss. Furthermore, regulation of autophagy and cellular metabolism by PIM2 were key mechanisms limiting effector cytokine production in CD8 T cells. We illustrated that PIM2 modulates VPRBP/EZH2 phosphorylation, inhibits EZH2 activity, and impairs memory-like phenotype of CD8 T cells. The suppressive role of PIM2 was extended in human CD8 T cells, in that inhibition of PIM2 improved activity of melanoma-specific TCR T cells and CD19 CAR T cells.

We demonstrated that *Pim2* deficiency enhanced cytokine secretion and effector function of tumor antigen–specific CD8 T cells in the TME (Figures 2 and 3). Importantly, transfer of *Pim2*-KO CD8 T cells did not cause immune pathogenicity given the nonelevated cytokine levels produced by them in peripheral lymphoid organs (Figure 3D). Upon continuous tumor antigen stimulation, T cells further develop into terminally differentiated, short-lived, exhausted T cells, characterized by a reduced ability to proliferate and secrete effector cytokines (1). Here, we suggest that PIM2 may contribute to CD8 T cell exhaustion in the TME, which is supported by attenuated coinhibitory receptor expression, elevated cytokine secretion, and increased proliferation of CD8^+^ TIL loss of *Pim2* (Figure 3A and Figure 4D). Recent studies highlight the importance of Tpex (2) and memory-like T cells (4, 5) in replenishing the antitumor effector response and sustaining CD8 T cell responses to cancer. PIM2 adversely impacted T cell therapy by restricting the persistence of transferred CD8 T cells (Figure 4). Interestingly, *Pim2* deficiency had no impact on Tpex (PD-1^+^TCF1^+^) cells in tumors (data not shown), but rather increased memory-like T cells in lymphoid organs. Possibly, a high level of CD62L maintenance of *Pim2*-KO CD8 T cells after in vitro activation (Figure 1G and Figure 2B) may facilitate their homing to lymphoid organs, where they are differentiated into memory T cells with the secession of antigen stimulation and supportive cytokines, including IL-7 and IL-15 (57). Our data suggest that PIM2 may affect CD8 T cell differentiation dynamics after cellular therapy possibly through limiting memory T cell formation in the lymphoid organs and effector T cell differentiation in the tumor. However, further studies are needed to delineate the precise role of PIM2 in regulating different stages/subsets of CD8 T cells.

We illustrated that PIM2 directly binds with P62 and enhanced autophagy flux in CD8 T cells (Figure 6 and Supplemental Figure 7). Attenuation of autophagy is a key mechanism underlying enhanced effector function of *Pim2*-KO CD8 T cells (Supplemental Figure 8). Autophagy, a cellular process to break down and recycle damaged organelles, plays a key role in regulating CD8 T cell–mediated antiinfection and antitumor responses (58, 59). In contrast, autophagy negatively regulates the effector function of CD8 T cells through inhibiting glycolysis and degrading granzymes and perforin (37, 41). High potassium concentration in the TME induces autophagy in TILs and limits their effector function but preserves stemness (60). Amino acid transport, induced by antigen stimulation and inflammatory cytokines (IL-2, IL-12, and IL-18), serves as a checkpoint for autophagy regulation in activated CD8 T cells (41). In general, autophagy is induced to promote cell survival under stress conditions, such as nutrient deprivation or hypoxia. Therefore, deletion of *Atg5*, *Atg7*, or *Atg3* impaired T cell homeostasis and survival (61). Similarly, increasing autophagy by moderate ER stress in CD8 T cells enhanced antitumor function (62). Moreover, the expression of *Atg5* or *Atg7* in virus-specific CD8 T cells is crucial for their survival and formation of functional memory T cells (63). Thus, in stress conditions, such as in the TME, PIM2 may contribute to autophagy induction in TILs, affecting their differentiation trajectory by preventing effector function. Meanwhile, in lymphoid organs, *Pim2*-KO CD8 T cells had similar levels of ATG5 expression (data not shown) and maintained higher stemness features and memory differentiation than WT controls (Figure 4). The dynamics and anatomical characteristics of autophagy regulation in effector and memory T cell differentiation following cellular therapy remain to be elucidated.

Increased EZH2 phosphorylation on multiple sites and increased H3K27me3 expression in *Pim2*-KO Pmel cells showed that PIM2 negatively regulates EZH2 activity (Figure 7, A and B). Using the EZH2 methyltransferase activity inhibitor GSK126, we confirmed that increased EZH2 activity contributes to enhanced memory-like phenotype and effector cytokine production in *Pim2*-deficient CD8 T cells (Figure 7G). EZH2 is a catalytic subunit of polycomb repressive complex 2 that trimethylates lysine 27 on histone H3 (H3K27me3), leading to repression of genes modulating survival, memory formation, and effector function of CD8 T cells (64). EZH2^+^CD8^+^ T cells were associated with improved survival in ovarian cancer patients because of EZH2’s positive role in polyfunctionality and survival of human effector T cells (65). EZH2 is required for the terminal effector cell differentiation and secondary response of CD8 memory T cells during antiviral response (66, 67). Consistent with our finding, EZH2 enforces the memory program and antitumor immunity of CD8 T cells through epigenetically activating *Id3* while inhibiting *Id2*, *Prdm1*, and *Eome* genes (48). The activity of EZH2 is negatively regulated by AKT through phosphorylation at Ser_21 (48). Given that PIM kinases and AKT kinases share some common targets in promoting cancer cell survival (68), PIM2 may act together with AKT to suppress EZH2 activity in CD8 T cells. Consistent with EZH2’s suppressive role in autophagy through activating mTOR (53), lower autophagy in *Pim2*-KO CD8 was partially restored by EZH2 inhibitor (Supplemental Figure 9). We conclude that PIM2 serves as an upstream kinase to inhibit EZH2 activity, leading to impaired memory-like features, persistence, and antitumor immunity of CD8 T cells.

We observed that PIM2 plays a role in regulating VPRBP phosphorylation and expression (Figure 7). PIM2 interacted with VPRBP and *Pim2* deficiency attenuated phosphorylation but increased abundance of VPRBP in CD8 T cells (Figure 7). VPRBP, or DCAF1 (Ddb1-cullin4-associated factor 1), is a cellular protein functioning through ubiquitination and phosphorylation of target proteins. VPRBP was reported to phosphorylate and stabilize EZH2 in colon cancer cells (52). When VPRBP kinase activity was inhibited, H3K27me3 expression in *Pim2*-KO CD8 T cells was reduced to the levels of WT cells (Supplemental Figure 9), suggesting that VPRBP positively regulates EZH2 activity in CD8 T cells as well. VPRBP is required for the cell cycle entry of activated T cells through ubiquitination and destabilization of p53 (69). VPRBP is essential for T cell expansion and function during antiviral and autoimmune responses (69). In addition, VPRBP restrains cellular reactive oxygen species through interacting with glutathione-*S*-transferase P, thus preventing aging of Tregs (70). Our data indicate an important role of VPRBP in CD8 T cell response in cancer immunotherapy, highlighting the need for future study.

The roles of PIM kinases in T cell biology appear to be isoform specific and context dependent. We previously demonstrated that *Pim2* deficiency in donor T cells accelerated, whereas *Pim1/Pim3* double deficiency in donor T cells attenuated GVHD severity, indicating a distinct role of PIM2 in regulating T cell allogeneic response (11). We also reported that *Pim1/2/3* triple deficiency in T cells promotes antitumor ability primarily by reducing glycolysis and increasing memory T cell differentiation in adoptive T cell therapy (10). Similarly, PIM3 was shown to promote hypoxia-induced dysfunction of CAR T cells through enhancing glycolysis and impairing memory formation (71). A pan-PIM kinase inhibitor, AZD1208, improved antitumor efficacy of T cells in both Pmel (10) and CAR T models (71). Conversely, PIM1 is required for CD8 effector function in antiviral response (72). PIM1 interacts with NF-κB to drive EOME expression and memory T cell maintenance during infection (73). In contrast, our study reveals that PIM2 impairs glycolysis and effector cytokine production and that PIM2 modulates EZH2 activity and impairs memory-like features in CD8 T cells. Collectively, these observations suggest that PIM kinases play nonredundant and sometimes opposing roles in regulating effector and memory T cell differentiation. Targeting PIM2 specifically may thus enhance effector function within the TME while supporting memory maintenance in lymphoid tissues, 2 key features for achieving durable and effective T cell–based immunotherapy.

We showed that targeting PIM2 through JP11646 treatment significantly enhanced murine and human CD8 T cell activity in controlling cancer (Supplemental Figure 11 and Figure 8). PIM kinases were identified as drivers of many malignancies, including solid tumors and hematological cancers (9). Therefore, pan inhibitors for PIM kinases have been developed and are in clinical trials for treatment of various cancer types, including B cell lymphoma, multiple myeloma, and prostate cancer (74, 75). However, so far, pan PIM inhibitors have not been effective, primarily due to toxicity, highlighting the need for targeting a specific isoform of PIM kinases that is safer and more effective. JP11646, a more selective PIM2 isoform inhibitor, has been shown to be effective in controlling cancer growth through PIM2 degradation in cancer cells (15). These findings together with ours support the development of PIM2 isoform–specific inhibitors for cancer treatment in which targeting PIM2 would promote T cell antitumor immunity while directly inhibiting malignant cells.

PIM2 kinase was reported to suppress human Th17 differentiation (76) and promote Treg expansion in rapamycin treatment (17); however, how PIM2 regulates human CD8 T cells remains unknown. To our knowledge, we are the first to show that PIM2 negatively impacts human CD8 T cells and that genetic or pharmacological inhibition of PIM2 markedly improves their activation, cytokine production, and cytolytic function (Figure 8 and Supplemental Figure 12). Currently, impaired persistence, limited expansion, and effector function of T cells contribute to failed CAR T therapy and TIL therapy (77, 78). Therefore, targeting PIM2 by CRISPR/Cas9 gene silencing in engineered T cells or use of a PIM2 inhibitor may represent a promising strategy to improve cancer immunotherapy through enhancing CD8 T cell persistence and effector cytokine production.

In conclusion, our study reveals a key role of PIM2 in negatively regulating CD8 T cell antitumor immunity. We present a model in which PIM2 interacts with P62 to modulate the autophagy pathway, thereby suppressing metabolic activity and effector cytokine production in CD8 TILs. In addition, PIM2 influences the VPRBP/EZH2 signaling axis to limit memory-like features in CD8 T cells in the lymphoid organs. Inhibition of PIM2 improved antitumor immunity mediated by both murine and human CD8 T cells. Because PIM2 has been shown to promote survival and proliferation of various cancer cells (12–15), it is a promising target for improving cancer therapy through enhancing effector function and persistence of CD8 T cells and inhibiting tumor growth. Thus, silencing or inhibition of PIM2 has the potential to enhance the efficacy of diverse cancer immunotherapy strategies. This work highlights the need to develop PIM2-isoform specific inhibitors with low toxicity before the concept can be applied to cancer immunotherapy in clinic.

## Methods

### Sex as a biological variable.

Both female and male mice were used in genetic and pharmacological experiments in melanoma and leukemia cancer models and only female mice in the breast cancer model.

### Experimental mice.

Female and male C57BL/6 (B6) Ly5.1 (H-2^b^, CD45.1), B6.Ly5.2 (H-2^b^, CD45.2), and FVB (H-2^q^) mice were purchased from Charles River Laboratories. Pmel (stock 005023), CD4Cre (stock 022071), Rag1^–/–^ (stock 008449), LC3 reporter (stock 027139), and NSG (NOD.Cg-*Prkdc^scid^*Il2rg*^tm1Wjl^*/SzJ; stock 005557) mice were purchased from The Jackson Laboratory. *Pim2*-KO mice in FVB background were a gift from Anton Berns (79). *Pim2*-KO FVB mice were crossed with HER-2/neu transgenic mice provided by Elizabeth M. Jaffee (25, 26) to generate antigen-specific T cells against HER-2/neu-expressing NT2.5 breast cancer cells. *Pim2*-KO FVB mice were crossed into B6 background 12 times to generate *Pim2*-KO B6 mice, which were then crossed with Pmel mice to generate T cells specific for melanocyte lineage-specific antigen gp100. All strains were maintained in a specific pathogen-free facility at an American Association for Laboratory Animal Care–accredited Animal Resource Center at the Medical College of Wisconsin (MCW).

### Generation of Pim2-KI mice.

We developed dual recombinase-responsive KI mouse strains, in which PIM2 was conditionally expressed at the Rosa26 locus after Cre- and Flp-mediated recombination (R26-CAG-LoxP-PIM2). The vector is composed of a ubiquitous CAG promoter (a combination of the chicken β-actin promoter and the cytomegalovirus immediate-early enhancer) as well as LoxP-flanked primary and Frt-flanked secondary stop cassettes. The transgene construct was inserted by homologous recombination into the Rosa26 locus in mouse embryonic stem cells. The T cell–specific *Pim2*-KI strain was generated by crossing *Pim2*-KI mice with CD4-Cre strain.

More details of the adoptive T cell protocol, allo-HCT, T cell purification, flow antibodies, Seahorse assay, Western blot, cell lines and reagents, RNA-seq, transmission electron microscopy, proteomics, T cell transduction, and CRISPR/Cas9 gene silencing can be found in Supplemental Methods.

### Statistics.

Statistical analyses were performed using GraphPad Prism 10. Data are shown as mean ± SEM. Survival data were analyzed using the log-rank (Mantel-Cox) test. Longitudinal tumor growth was analyzed using a mixed-effects model (REML) with genotype and time as fixed effects and mouse as a random effect, with Greenhouse-Geisser correction. For analyses involving a single experimental factor, comparisons between 2 groups were performed using an unpaired 2-tailed Student’s *t* test, whereas comparisons among more than 2 groups were performed using 1-way ANOVA with Tukey’s or Bonferroni post hoc test, as indicated in the figure legends. A *P* value < 0.05 was considered statistically significant.

### Study approval.

Animal experiments were conducted in accordance with protocols approved by the MCW IACUC.

### Data availability.

All data associated with the study are present in the main text or the Supplemental Methods. The values corresponding to all data points shown in graphs and values behind any reported means are available in the Supporting Data Values file. RNA-seq data sets have been deposited in the Sequence Read Archive database with accession number SAMN53298971. The proteomics raw data files have been deposited in the PRIDE Proteomics Database with project number PXD059837.

## Author contributions

YW participated in experimental design; performed research; collected, analyzed, and interpreted data; performed statistical analysis; and drafted and revised the manuscript. LT participated in experimental design; performed research; and collected, analyzed, and interpreted data. AP performed research, collected and analyzed data, performed statistical analysis, and genotyped mice. RA performed sgRNA deletion of *PIM2* in CAR T cells and examined their in vivo function. QC performed research and interpreted data. WC participated in experimental design of CRISPR/Cas9 silencing of PIM2 in human T cells. MIN provided TIL 1383I TCR and CD19 CAR plasmids. LEB performed the phosphoproteomics assay and analysis. CWL performed statistical analysis and figure generation for phosphoproteomics data. SM participated in experimental design and revised the manuscript. ASK provided *Pim2*-KO and generated *Pim2*-KI mice. XZY designed research, interpreted data, performed statistical analysis, and revised the manuscript.

## Funding support

This work is the result of NIH funding, in whole or in part, and is subject to the NIH Public Access Policy. Through acceptance of this federal funding, the NIH has been given a right to make the work publicly available in PubMed Central.

- NIH grants R01CA258440 and R01HL163584 (to XZY).
- Institutional start-up funds from The Cancer Center and Department of Microbiology & Immunology, MCW (to XZY).
- Supported in part by the MCW Cancer Center Shared Resource.
- Supported in part by the MCW Flow Cytometry and Cell Sorting Unit.

## Supplementary Material

Supplemental data

Unedited blot and gel images

Supporting data values

## Version 1 01/27/2026

In-Press Preview

## Version 2 03/16/2026

Electronic publication
