# Supplementary material for: Targeting PIM2 improves antitumor immunity through promoting effector function and persistence of CD8 T cells
Source: J Clin Invest. 2026 Jan 27;136(6):e192928. doi: 10.1172/JCI192928 (PMC12987625; doi:10.1172/JCI192928)
Supplement: Supplemental data [file jci-136-192928-s100.pdf]

**Supplementary Materials**

**Title: Targeting PIM2 Improves Antitumor Immunity through Promoting Effector Function and Persistence of CD8 T cells**

**Authors:** Yongxia Wu[1,2*§], Linlu Tian[1§], Allison Pugel[1], Reza Alimohammadi[1], Qiao Cheng[1], Weiguo Cui[3], Michael I. Nishimura[4], Lauren E. Ball[5], Chien-Wei Lin[2], Shikhar Mehrotra[6], Andrew S Kraft[7], and Xue-Zhong Yu[1,2,8*]

**Materials and Methods**

**Fig. S1 to Fig. S12 for multiple supplementary figures.**

## Materials and Methods

**Adoptive T-cell protocol.** B16F10 tumor were subcutaneously (s.c.) injected on the left flank or intravenously (i.v.) infused at $0.2\times10^6$/mouse in Ly5.1 B6 mice. On day 6 or 3 following B16F10 cell inoculation by s.c. or i.v., respectively, tumor-bearing B6 mice received sublethal dose of total body irradiation (TBI) at 600cGy using an J.L. Shepherd Mark I $Cs^{137}$ irradiator. Then 24 hours after irradiation, they were either kept untreated or adoptively transferred with $2\times10^6$ pre-activated, with gp100 peptide at 500ng/ml for 3 days, WT or *Pim2*$^{-/-}$ Ly5.2$^+$CD8$^+$ Pmel cells. To establish a breast cancer model, a mixture of 50μl of NT2.5 cells ($2\times10^6$/mouse) plus 50μl Matrigel was injected into the 4$^{th}$ mammary pad of female Thy1.1 FVB mice. After 14 days, these NT2.5-bearing mice were adoptively transferred with $1\times10^6$ pre-activated, WT or *Pim2*$^{-/-}$ Clone 100 TCR-Tg Thy1.2$^+$ CD8$^+$ T cells (100ng/ml RNEU$_{420\text{-}429}$ peptide for 3 days). Tumor growth in diameter was measured with a vernier caliper 2 times a week. In xenograft model, NSG mice were i.v. injected with $1\times10^6$ GFP$^+$ Raji cells and $1\times10^6$ CAR$^+$ T cells per mouse 3 days after tumor infusion. Where indicated, JP11646 or vehicle (DMSO) was administrated i.p. at 7.5 mg/kg twice per week for 4 weeks after ACT.

**Hematopoietic cell transplantation (HCT).** B6 mice (8–10 weeks of age) were conditioned with TBI at 1200 cGy. The recipients were i.v. injecfted with $1\times10^6$ C1498$^{luc}$ leukemic cells and $5\times10^6$ bone marrow (BM) cells from WT B6 mice with or without $7.5\times10^6$ CD25-depleted splenocytes from WT or *Pim2*$^{-/-}$ B6 mice within 24 h after TBI. In allogeneic HCT, lethally irradiated B6 mice were i.v. injected with $5\times10^6$ BM cells with or without WT or *Pim2* KO FVB T cells at $1\times10^6$ per mouse.

**T-cell purification.** T cells were purified from spleen and lymph nodes by negative selection using magnetically labeled biotin-conjugated antibodies against CD45R (clone RA3-B2), CD49b (clone DX5), CD11b (clone M1/70), and Ter-119 (clone Ter-119); all antibodies were from eBioscience and anti-biotin microbeads and LS column were from Miltenyi Biotec. In some experiment, CD25$^+$ cells were removed from splenocytes using anti-CD25 biotin-conjugated antibody (PC61.5).

**Cell staining and flow cytometric analysis.** Standard flow cytometric surface staining was performed. The flow cytometry antibodies used were as follows: CD4 (RM4-5, BD), CD8 (53-6.7, BD), CD45.2 (104, BD),CD45.1 (A20, BD), IFNγ (XMG1.2, eBioscience), IL-2 (JES6-5H4, BD), TNFα (MP6-XT22, BD), CD44 (IM7, eBioscience), CD25 (PC61.5, BD ), CD62L (MEL-14, BD), LAG3 (C9B7w, Invitrogen), PD-1 (J43, BD), Ki67 (B56, BD), TCF1 (S33-966, BD), LY108 (13G3, BD), ATG5 (177.19, eBioscience), CX3CR1 (SA011F11, Biolegend), CD127 (clon A7R34, BD), KLRG1 (2F1, BD), SCA-1 (D7, Biolegend), TCRvb13 (MR12-3, BD), Streptavidin-PE-Cy7 (BD), and Streptavidin-APC-Cy7 (BD). For CFSE staining, prewarmed cells were stained with CFSE (BD Horizon) at 0.7 μM for 7 min, and immediately stopped with 10% FBS RPMI 1640 medium. For metabolic panels, 100μM 2NBDG (11046, Cayman Chemical), 6μM BodiPy (D-3823, Invitrogen), 25nM Mito-tracker (M7512, ThermoFisher) or 200nM TMRM (T668, ThermoFisher) was incubated with cells at 37°C for 20 mins. Stained cells were analyzed using LSR II (BD Biosciences, San Jose, CA), Cytek (Cytek Biosciences, San Diego, CA) and FlowJo (TreeStar, Ashland, OR).

**Seahorse assay**. The metabolic profile of single-cell suspensions was determined using a Seahorse XF96 Analyzer (Seahorse Bioscience). Briefly, T cells ($0.3\times10^6$/well) were re-suspended in glucose-free medium and attached to tissue culture plates for 30 minutes using Cell-Tak (BD Biosciences, catalog 354240). The basal conditions and following injection of following pharmacologic compounds, including glucose (10mM), oligomycin (1μM), and 2-DG (100mM) for ECAR; Oligomycin (10μM), FCCP (10μM) and antimycin/rotenone (20μM) for OCA were measured using Seahorse XFe/XF Analyzer. Data were analyzed using the Seahorse Wave software.

**Western blot and immunoprecipitation**. Whole-cell lysates were collected using lysis buffer supplemented with the protease inhibitor cocktail solution (MCL-1, Sigma-Aldrich), electrophoresed in a precast polyacrylamide gel and blotted onto PVDF membranes (Bio-Rad Laboratories Inc.). After blocking with 5% nonfat dry milk, the membranes were incubated with primary antibody specific for PIM2 (1D12, Santa Cruz Biotechnology), PIM2 (5D5, Origene), H3K27Me3 (C36B11, Cell Signaling), EZH2 (11/EZH2,

BD), VPRBP (1612-1-AP, ThemoFisher), LC3b (#2775, Cell Signaling), P62 (#5114, Cell Signaling), β-Actin (#4967, Cell Signaling),or GAPDH (D16H11, Cell Signaling) overnight at 4°C, followed by washing and addition of a HRP-conjugated goat-anti-rabbit or goat-anti-mouse secondary antibody for 1 hour. Blots were treated with ECL western blotting substrate (Thermo Fisher Scientific). For immunoprecipitation experiment, PIM2 (1D12, Santa Cruz) or mouse IgG1 (sc-3877, Santa Cruz) were added into cell lysate (1:100) for 6 hours with constant rocking at 4°C, then pre-cleared protein A/G beads were added and incubated with constant rocking overnight at 4°C. Beads were washed 3-4 times then boiled in laemmli buffer. Western blots for P62, EZH2, VPRBP and PIM2 were performed.

**Cell lines and Reagents.** NT2.5 breast cancer cell line was kindly provided by Dr. Sgouros (1). B16F10, C1498 and Raji cells were purchased from ATCC. These tumor cell lines were expanded *in vitro* in complete RPMI medium before tumor inoculation in mice. JP11646 (PC-63142, ProbeChem), B32B3 (294193-86-5, Sigma), GSK126 (HY-13470, MCE) were dissolved in DMSO and metformin (317240, Sigma) was dissolved in PBS. For *in vivo* administration, JP11646 or DMSO was administrated i.p. at 7.5 mg/kg twice per week for 4 weeks after ACT.

**Single-cell RNAseq.** CD8 T cells from spleen and TDLNs were purified using CD8 TIL beads followed by flow sorting of live $CD8^+$ cells and single-cell RNA sequencing (scRNA-Seq). The libraries were generated using Chromium Single Cell 3′ v.2 and sequenced using the NextSeq 500 System (Illumina) to a depth of about 300 million reads per library with $2 \times 50$ read length. Cell Ranger (10x Genomics) were used to demultiplex the sequencing data and generate gene-barcode matrices, respectively. All scRNA-seq analyses were performed in R using the package Seurat. The differential expressed genes (DEGs) were determined using FindMarkers package (Wilcoxon rank-sum test) and Pathway enrichment analysis were perform using Metascape (2). Genes with an adjusted P-value <0.05 found by FindMarkers were assigned as differentially expressed. The data sets have been deposited to Sequence Read Archive (SRA) database with access number SAMN53298971.

**Transmission electron microscopy.** Purified T cells from WT or *Pim2* KO mice were stimulated with plate bounded anti-CD3/CD28 at 2μg/ml for 3 days. Detection was perform using Transmission electron microscopy (TEM). Briefly, cells were fixed in 2.5% glutaraldehyde, 100 mM sodium cacodylate buffer pH 7.2, post-fixed in 1% osmium tetroxide on ice for 1 hour, dehydrated in a graded methanol series, and embedded in EPON 812 (EMS). Sixty nanometer sections were stained with uranyl acetate and Reynolds lead citrate and examined with a JEOL 2100 transmission electron microscope.

**Proteomics and Phosphoproteomics.** WT or *Pim2* KO Pmel splenocytes (5 mice each group) were stimulated with 750ng/ml gp100 peptides for 3 days (>90% cell were CD8 T cells). Live cells were enriched using Ficoll and then re-stimulated with gp100 at 1000 ng/ml in plain RPMI for 1h in 37°C and stop with ice-cold PBS containing 0.5 mM EDTA and 2 mM Na3VO4. Cells were lysed in 9 M urea, 50 mM Hepes pH 8.5 amended with Halt phosphatase inhibitor cocktail, EDTA free-Halt protease inhibitor cocktail, and Thermo Universal Nuclease by passing through a 22-gauge needle. Protein concentration was measured by BCA assay. Proteins (1.5mg) were reduced in 5 mM dithiothreitol, alkylated in 10 mM iodoacetamide followed with adding 50 mM Hepes pH 8.5, Lys-C (Waco) for 2 hr at room temperature and trypsin (Sigma-Aldrich) digestion overnight at 37 °C. Digestion was quenched by acidifying the samples with formic acid. The resulting peptides were desalted with SepPak columns (Waters), dried by vacuum centrifugation, redissolved in 0.1% formic acid, and the concentration determined using the Quantitative Colorimetric Peptide Assay Kit (Pierce).

Peptide aliquots were dried, redissolved in 200 mM Hepes pH 8.5, and labeled with Tandem Mass Tag labels (TMT10plex, Thermo Scientific). The TMT labeled peptides were combined and fractionated by high pH reversed phase chromatography using an Agilent 1100 HPLC system with a Zorbax 300Extend C18 column. Fractions were acidified, dried, and combined by concatenating into 12 fractions. Peptides were desalted using SepPak columns and dried. Phosphopeptides from each fraction were sequentially enriched twice using High-Select $TiO_2$Phosphopeptide Enrichment Kit (Thermo Scientific) and then enriched twice using High-Select Fe-NTA Phophopeptide Enrichment Kit (Thermo Scientific).

Phosphopeptides were desalted by Stage Tip and analyzed by LC-MS/MS. Peptides were separated on an Acclaim PepMap RSLC C18 (50 cm x 75 µm) column. The precursor scan was acquired in the orbitrap at 120K resolution with a mass range of 400-1500 m/z and a maximum injection time of 50 ms. Peptides were fragmented by higher energy collisional dissociation (HCD). The TMT reporter ions in the MS2 scan were used for quantitation. To assess global changes in protein expression, an aliquot from each of the 12 fractions was analyzed on the Orbitrap Fusion Lumos MS as described above with synchronous precursor selection (SPS)-MS3 implemented. MS2 spectra were acquired using collision induced dissociation (CID) with a 35% collision energy and 1.2 isolation window. The 10 most intense fragments from the MS2 scan were selected for MS3 fragmentation (HCD collision energy 65%, isolation window of 1.3 Da). The reporter ions from the MS3 scan were used for quantitation at the protein level. The data was deposited into PRIDE Proteomics Database with Project # PXD059837.

**Data processing of proteomics.** The raw data were searched in MQ 2.0.1.0 (Max Plank Institute) against a reviewed murine protein database downloaded from UniProt (3/15/2023 with 17,141 entries) and a decoy database. Fixed modifications included TMT labels on lysine and peptide N-termini as well as carbamidomethylation of cysteines. Oxidation of methionine and protein N-terminal acetylation were used as variable modifications. An FDR <0.01 was required for identification at the protein, PSM, and site levels. The minimum peptide length was 7 and minimum score for modified peptides was 40 with a delta score of 6. For analysis of differentially expressed proteins, the protein groups text file was processed and statistically evaluated in Perseus (v. 1.6.15.0) (Max Plank Institute). Protein hits to the decoy database, common cell culture contaminants, and proteins only identified by a single modified peptide were removed leaving 5,704 proteins. The corrected, reported ion intensities were log2 transformed and the data were filtered to retain entries with 3 quantitative values in at least one of the groups, wild type or knock out. Missing values were imputed from a normal distribution and each column (label) normalized by subtracting the median. An arbitrary value of 20 was added to each value to avoid negative intensities. The entries were annotated with GO terms and Reactome Pathways. A two sample Student's t-test was performed to

compare the protein intensities and a permutation based false discovery rate of 0.05 was used as a threshold for differentially expressed proteins. For the phosphoproteomic dataset, the analysis was performed as above with the following modifications. Phosphorylation was included as variable modification of serine, threonine, and tyrosine. The phosphosite text file was filtered, annotated, and statistically evaluated in Perseus. Entries were annotated with linear motifs, known sites, and other terms from the PhosphoSite Plus database (downloaded 9/9/2022) (Hornbeck). Phosphosites with >75% localization probability were retained. 18,359 putative phosphorylation sites from 4,449 proteins were identified.

Differentially expression (DE) analysis for each individual phosphosite between WT and KO (figure 7A) was conducted by two-sample t-test. Meta-analysis Fisher's method was then used to aggregate p-values from all phosphosites within the same protein. Proteins with meta-analyzed FDR less than 0.05 were considered statistically significant. Top 100 DE proteins reported to be regulators of T cells were selected and heatmap was generated by R package ComplexHeatmap. Pathways related to CD8 T-cell exhaustion, effector and memory differentiation were downloaded from MsigDB c7 gene lists. To quantify the overall abundance level of DE proteins in each pathway, we first calculated protein-level abundance by taking average across individual phosphosites within each DE protein. Average abundance level of each pathway was then calculated across DE proteins within and subtracted by the aggregated abundance level of randomly selected background proteins.

**Transduction of T cells**. CD19CAR and Til1383i vectors were produced as described previously (3-5). For viral transduction step, human PBMCs (Research blood components, LLC) were thawed, rested overnight, and stimulated with 50ng/ml OKT3 for 2 days in IMEM complete medium. Cells were washed and resuspend at $1\times10^6$/ml in fresh medium. Transduction were performed in retronectin-pre-coated plates (30ug/ml) and spun at 2000g in 32°C for 2 hours. Cells were cultured and expanded in 300IU/ml hIL-2 for 6 days.

**CRISPR/Cas9 RNP transfection.** Ribonucleoprotein (RNP) electroporation was performed with human PBMC using EasySep immunomagnetic negative selection kits from STEMCELL. Briefly, Cas9 (Alt-R

S.p. Cas9 Nuclease, IDT) and sgRNAs (Synthego) were combined and incubated at RT for 10 min. For each target, three sgRNAs was used to increase knockout efficiency. Electroporation was performed using the 4D-NucleofectorTM 4 Core Unit and P3 primary cell 4D-NucleofectorTM5 X kit S with program DN100. Following the electroporation, cells were kept in an incubator for 10 min at 37°C. The cells were activated with anti-CD3 and anti-CD28 for 3 days for flow test or further transduced with CD19CAR for *in vivo* function test in Raji-baring NSG mice as described above.

**References**


1. Song H, Shahverdi K, Huso DL, Wang Y, Fox JJ, Hobbs RF, et al. An immunotolerant HER-2/neu transgenic mouse model of metastatic breast cancer. *Clin Cancer Res.* 2008;14(19):6116-24.
2. Zhou Y, Zhou B, Pache L, Chang M, Khodabakhshi AH, Tanaseichuk O, et al. Metascape provides a biologist-oriented resource for the analysis of systems-level datasets. *Nat Commun.* 2019;10(1):1523.
3. Wang SY, Moore TV, Dalheim AV, Scurti GM, and Nishimura MI. Melanoma reactive TCR-modified T cells generated without activation retain a less differentiated phenotype and mediate a superior in vivo response. *Sci Rep.* 2021;11(1):13327.
4. Wang SY, Scurti GM, Dalheim AV, Quinn S, Stiff PJ, and Nishimura MI. Nonactivated and IL-7 cultured CD19-specific CAR T cells are enriched in stem cell phenotypes and functionally superior. *Blood Adv.* 2024;8(2):324-35.
5. Chatterjee S, Daenthanasanmak A, Chakraborty P, Wyatt MW, Dhar P, Selvam SP, et al. CD38-NAD(+)Axis Regulates Immunotherapeutic Anti-Tumor T Cell Response. *Cell Metab.* 2018;27(1):85-100.e8.

**Supplementary figures**

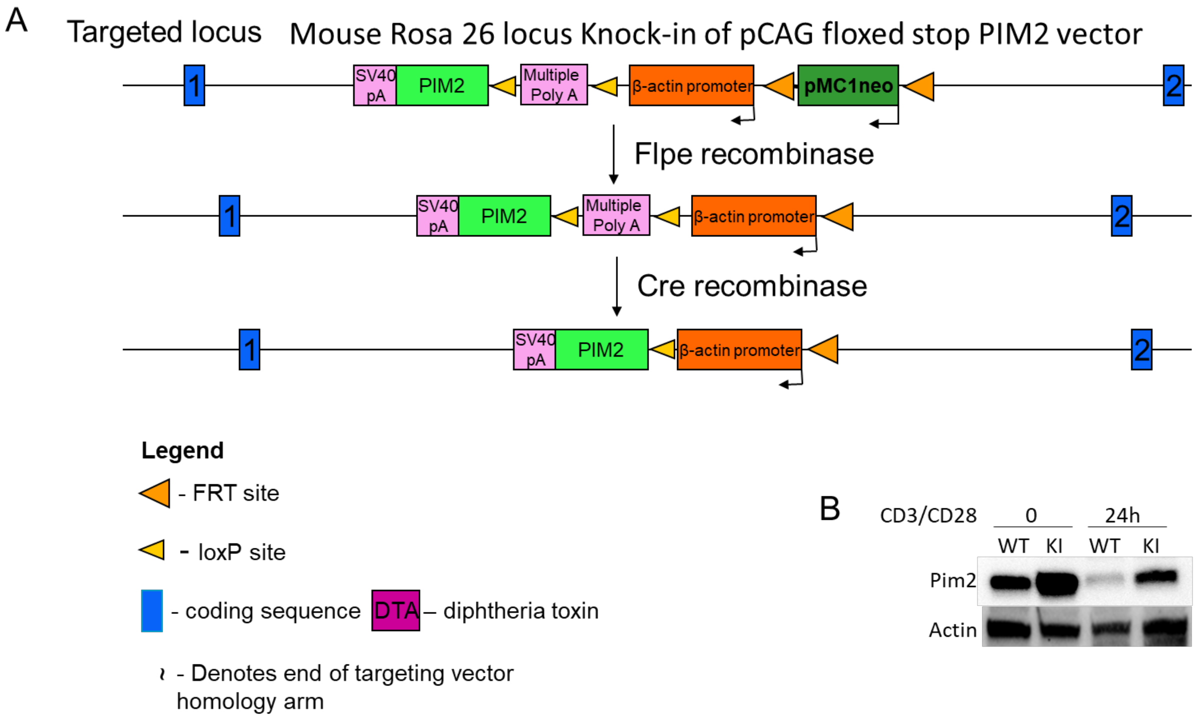


**Figure S1. Generation of *Pim2* KI mice. (A)** The diagram of mouse generation with *Pim2* KI specifically in T cells (refer M&M for details). **(B)** T cells were purified from WT, *Pim2* KI or *Pim2* KO mice. PIM2 and Actin expression were detected by western blotting. Data represent 3 independent experiments.

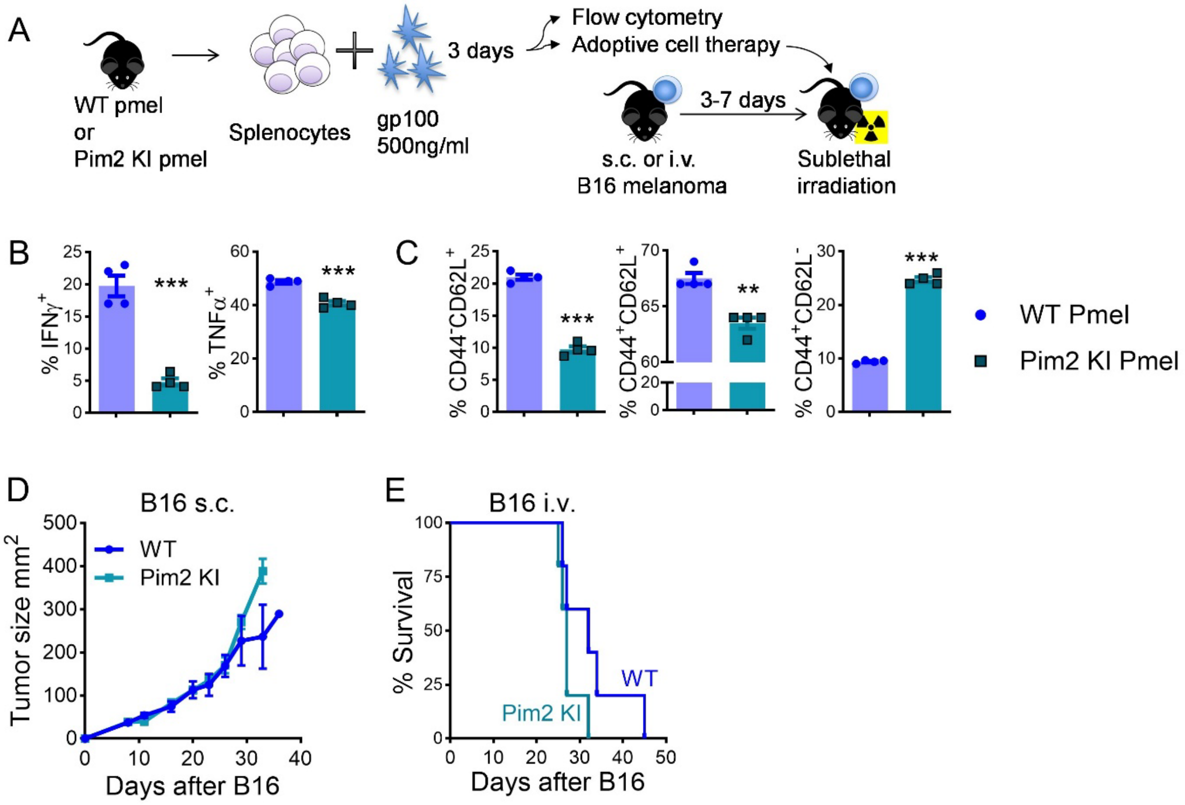


**Figure S2. Overexpression of PIM2 reduces anti-tumor response of CD8 T cells.** **(A)** Ly5.1 B6 mice were s.c. infused with B16F10 tumor on the flank, followed with sublethal irradiation at 600cGy and adoptive transfer of gp100 peptide pre-activated WT or *Pim2* KI CD8$^+$ Pmel T cells on day 7. **(B-C)** The phenotype of CD8$^+$ Pmel cells post gp100 peptide stimulation were evaluated by flow cytometry. Data represent 2 independent experiments. **(D)** Tumor growth was monitored. WT n=5 and KO n=5. **(E)** Ly5.1 B6 mice were i.v. injected with B16F10, followed with sublethal irradiation and adoptive transfer of gp100 peptide pre-activated WT, *Pim2* KI pmel CD8 T cells on day 3. **(E)** Survival and melanoma growth monitored by bioluminescence imaging (BLI) are shown. WT n=5 and KO n=5. Data represent 2 independent experiments (E-G). Data were analyzed by two-tailed Student's t test (B, C), two-way ANOVA (D), and log-rank test for survival curves (E). Data plotted are mean ± SEM from biological replicates. *p<0.05, **p<0.01, ***p<0.001.

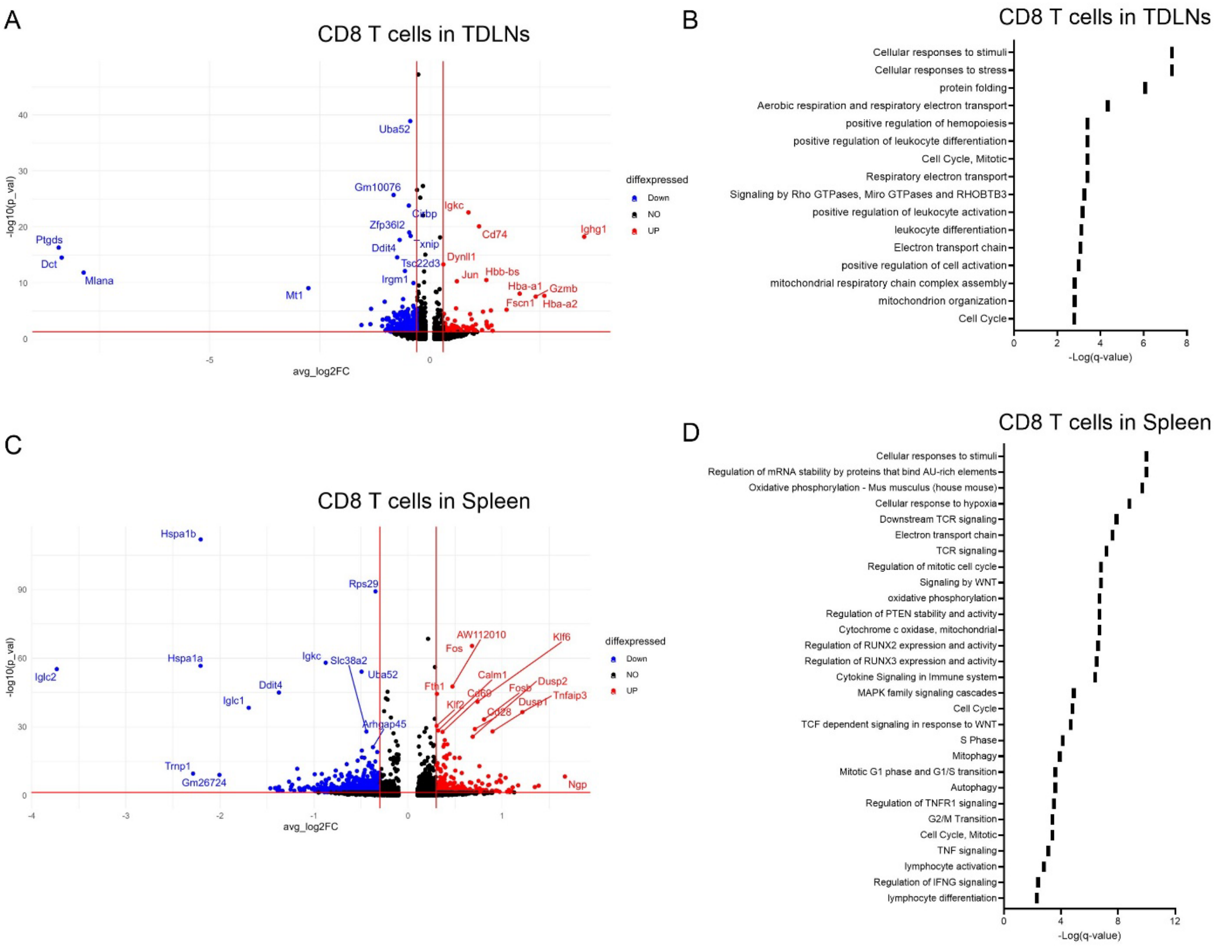


**Figure S3. Adoptive transfer of *Pim2* KO Pmel CD8 T cells promotes host CD8 T-cell activation.** Ly5.1 B6 mice were s.c. infused with B16F10 tumor on the flank, followed with sublethal irradiation at 600cGy and adoptive transfer of gp100 peptide pre-activated WT or *Pim2* KO pmel T cells on day 7. At day 21 post ACT, CD8 T cells from spleen and TDLNs were purified using CD8 TIL beads followed by flow sorting of live CD8$^+$ cells and single-cell RNA sequencing. Volcano plots present the differentially expressed genes (DEGs) in CD8 T cells isolated from **(A)** TDLNs and **(C)** spleen of tumor-bearing mice that received *Pim2* KO versus WT pmel cells. Pathway enrichment analysis was performed in those genes that were significantly upregulated in CD8 T cells from TDLNs **(B)** and spleen **(D)** of the recipients received *Pim2* KO than WT Pmel cells. Differential gene expression between cell populations was performed using the FindMarkers function (A, C). Pathway enrichment analysis was performed using Metascape (B, D).

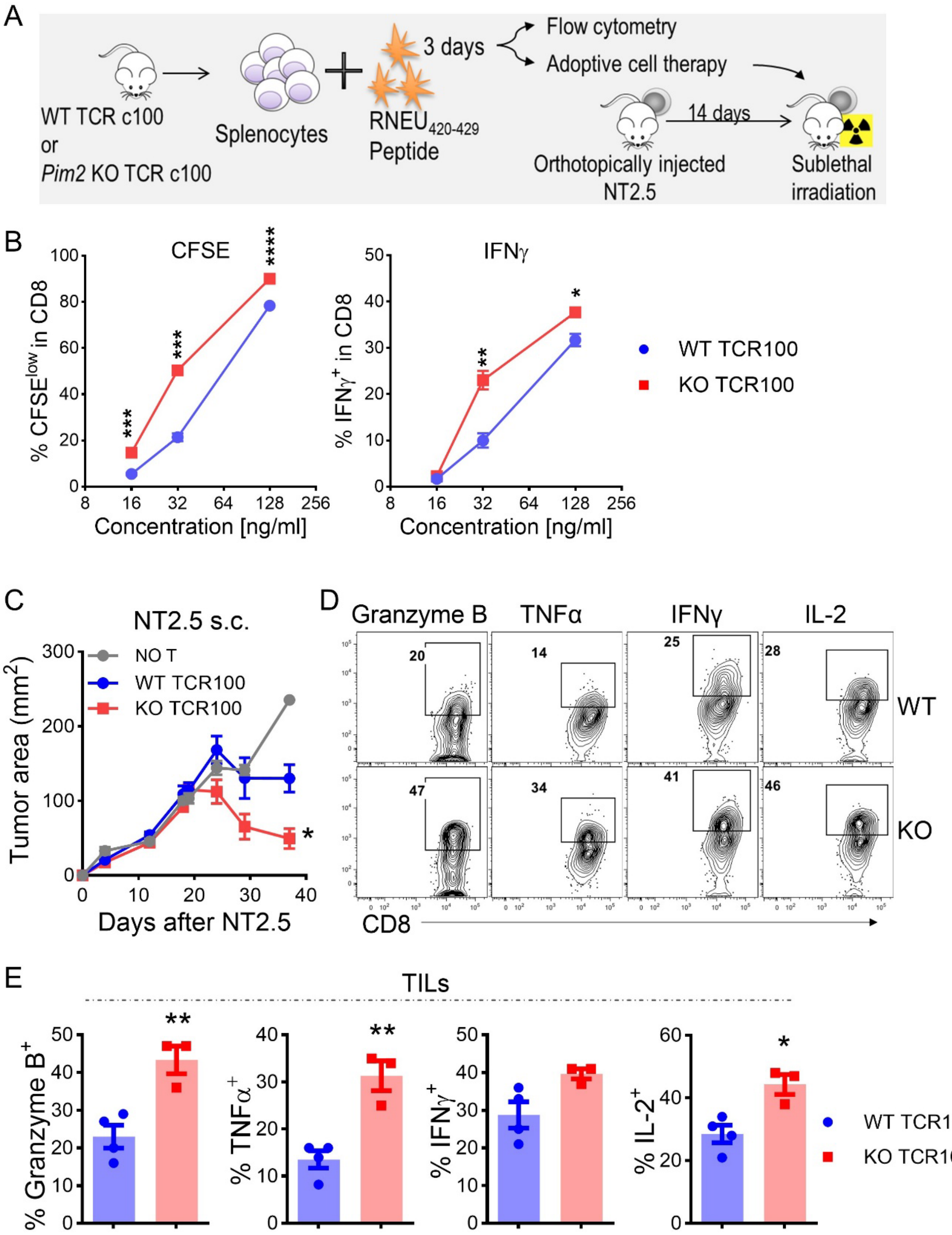


**Figure S4.** ***Pim2*** **deficiency enhances activation and effector function of tumor-antigen-specific CD8 T cells in controlling breast cancer. (A)** NT2.5 mammary cancer cells were injected under right mammary fat pads of Thy1.1$^+$ WT FVB mice. After tumor was established for 14 days, these tumor-bearing mice

were adoptively transferred with $RNEU_{420-429}$ peptide pre-activated WT or *Pim2*$^{-/-}$ Clone 100 TCR-Tg CD8 T cells (Thy1.2$^{+}$). **(B)** CFSE dilution and IFNγ production in WT or *Pim2* KO TCR clone 100 T cells post $RNEU_{420-429}$ stimulation were evaluated by flow cytometry. **(C)** Tumor growth was monitored. **(D-E)** At the end of experiment, TILs were isolated and cytokine expression in gated Thy1.2$^{+}$ CD8 T cells are shown. Data represent 2 independent experiments with WT n=9 and KO n=9. Data were analyzed by two-tailed Student's t test (E) and two-way ANOVA (B, C). Data plotted are mean ± SEM from biological replicates. $^{*}p<0.05$, $^{**}p<0.01$, $^{***}p<0.001$.

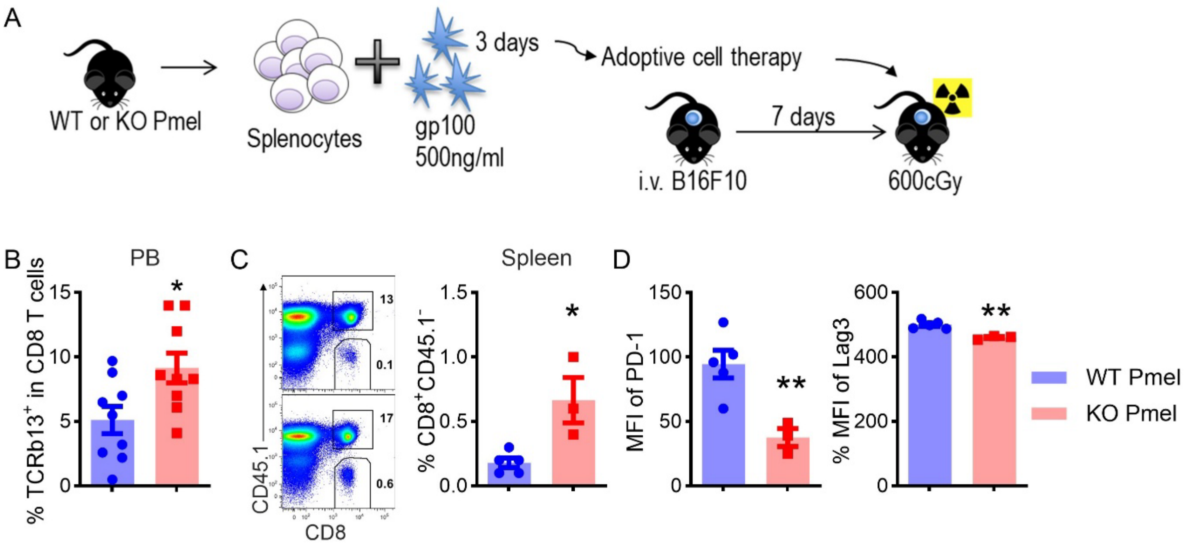


**Figure S5. CD8$^{+}$ *Pim2* KO Pmel T cells have decreased exhaustion phenotype and increased persistence in metastatic B16 model. (A)** Ly5.1 B6 mice were i.v. injected with B16F10, followed with sublethal irradiation at 600cGy and adoptive transfer of gp100 peptide pre-activated WT or *Pim2* KO CD8$^{+}$ Pmel T cells on day 7. **(B-C)** On day 21, frequencies of Pmel cells (Ly5.2+) in peripheral blood and spleen were examined by flow in gated live cells. **(D)** Expression of PD-1 and LAG3 on Pmel T cells from spleen are shown. Data represent 2 independent experiments with WT n=10, and *Pim2* KO n=10. Data were analyzed by two-tailed Student's t test and plotted as mean ± SEM from biological replicates. *p<0.05, **p<0.01, ***p<0.001.

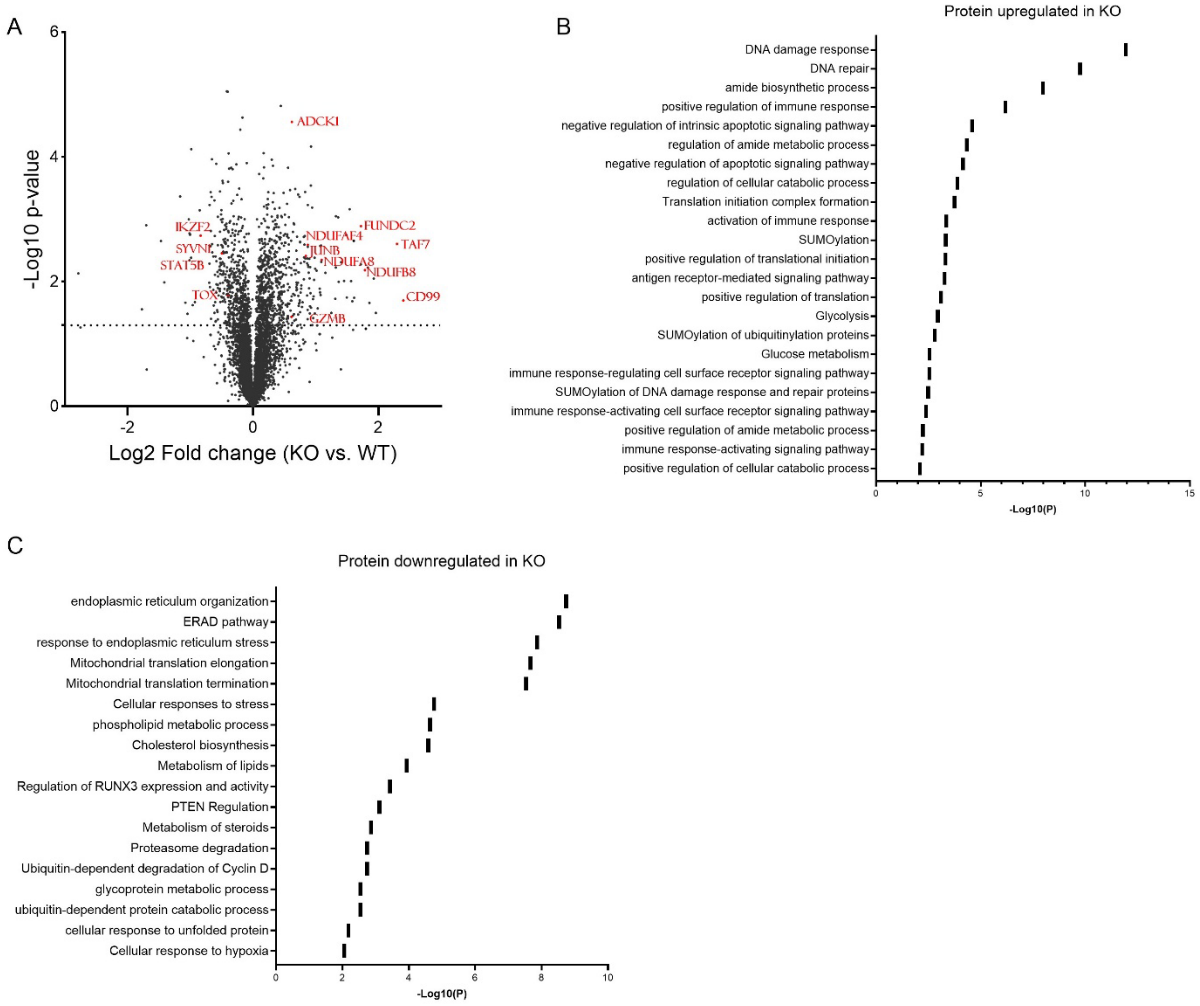


**Figure S6. PIM2 regulates expression of proteins related to cell metabolism and ER stress in CD8 T cells.** WT or *Pim2* KO pmel splenocytes were activated with 500ng/ml gp100 peptide for 3 days. These activated CD8 T cells were subjected to proteomics analysis. WT n=5 and KO n=5. **(A)** Volcano plots present the differentially expressed phosphoproteins between WT and *Pim2* KO Pmel cells. Pathway enrichment analysis was performed in the proteins that were significantly elevated in the **(B)** *Pim2* KO or **(C)** WT Pmel cells. WT n=3, and KO n=3. A two sample Student's t-test was performed to compare the protein intensities and a permutation based false discovery rate of 0.05 was used as a threshold for differentially expressed proteins (A). Pathway enrichment analysis was performed using Metascape (B, C).

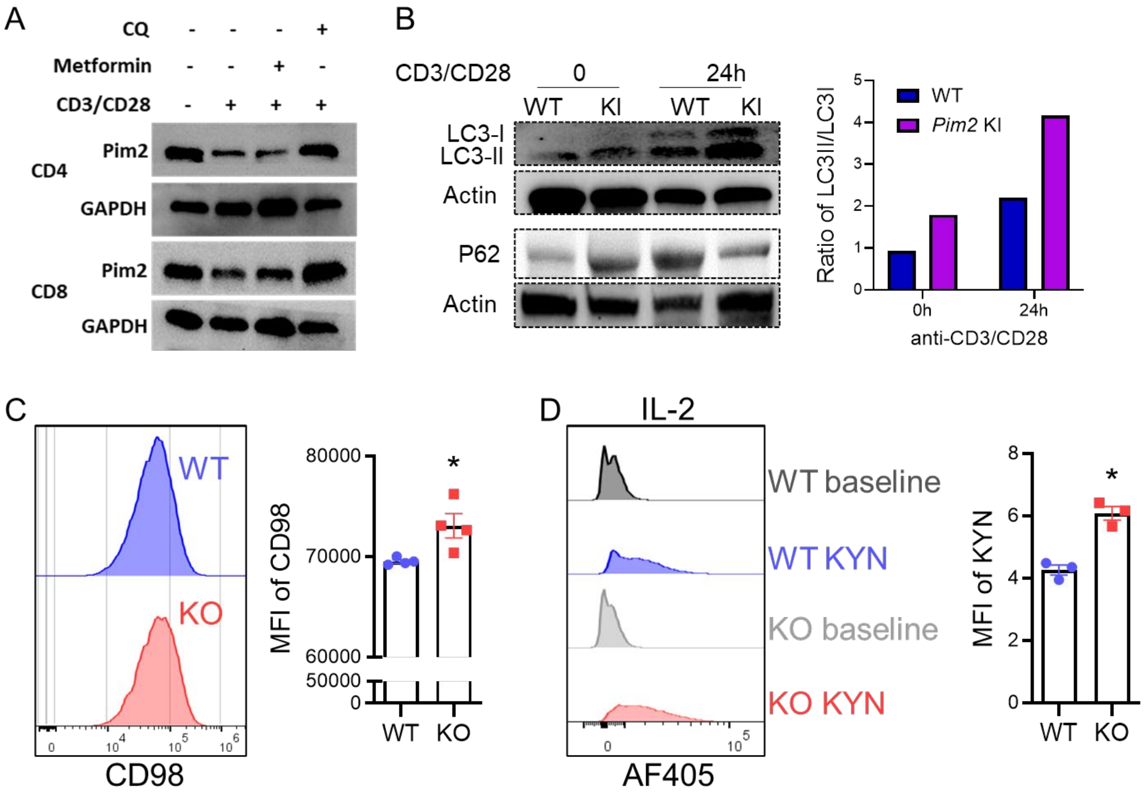


**Figure S7. PIM2 increases autophagic flux in T cells upon activation.** Pan T cells were isolated from B6 mice and stimulated with plate-bounded anti-CD3 (5μg/ml) and soluble anti-CD28 (2μg/ml) in the presence of chloroquine (CQ, 10μM) or metformin (100μM) for 48h. **(A)** PIM2 and **(B)** actin expression were examined by western blotting. (**C**) WT or *Pim2* KO pmel splenocytes were activated with 500ng/ml gp100 peptide for 3 days. CD98 expression gated on CD8 T cells are shown. (**D**) WT or *Pim2* KO pmel splenocytes were activated with 500ng/ml gp100 peptide for 3 days and followed with IL-2 (10ng/ml) culture for 3 days. Cells were incubated with pre-warmed kynurenine (KYN) at 400μM for 20min at 37°C and immediately fixed with 2% PFA. The 405nm laser and 450/50 BP filter (AF405) was used to detect KYN. A relative MFI of KYN is calculated by dividing the MFI of the stained samples by the MFI of the non-staining (baseline) controls. The experiment was repeated one more time. Data were analyzed by two-tailed Student's t test (C, D) and presented as mean ± SEM from technical replicates. *p<0.05.

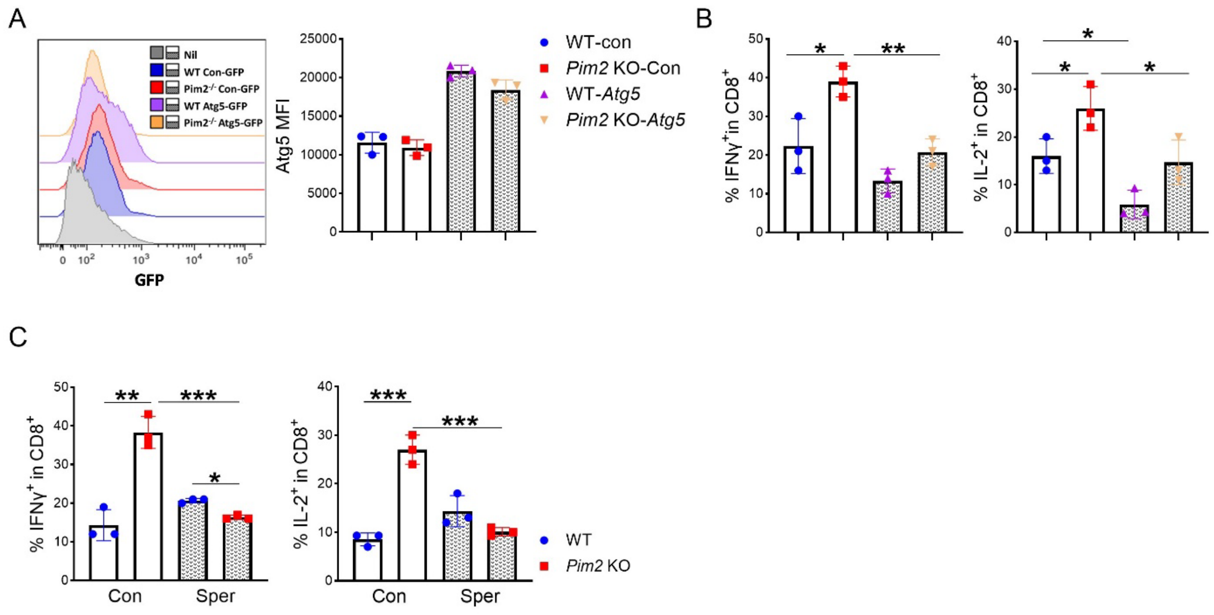


**Figure S8. Autophagy induction attenuates effector cytokine production in *Pim2* KO CD8 T cells. (A-B)** Purified T cells from WT or *Pim2* KO FVB mice were transduced with Atg5-GFP and activated by T-cell depleted allogeneic splenocytes *in vitro*. **(A)** ATG5 expressed was examined by flow 48h after transduction. **(B)** Percentages of IFNγ, and IL-2 expressing cells among gated CD8 T cells were measured by flow cytometry after 4-day culture. **(C)** Purified T cells from WT or *Pim2* KO FVB mice were activated by allogeneic APCs *in vitro* in the presence of DMSO or spermidine (10μM) for four days. Percentages of IFNγ, and IL-2 expressing cells among gated CD8 T cells were measured by flow cytometry after 4-day culture. Figure A-C, data represent 2 independent experiments. Data were analyzed by one-way ANOVA and presented as mean ± SEM from technical replicates. *p<0.05, **p<0.01, ***p<0.001.

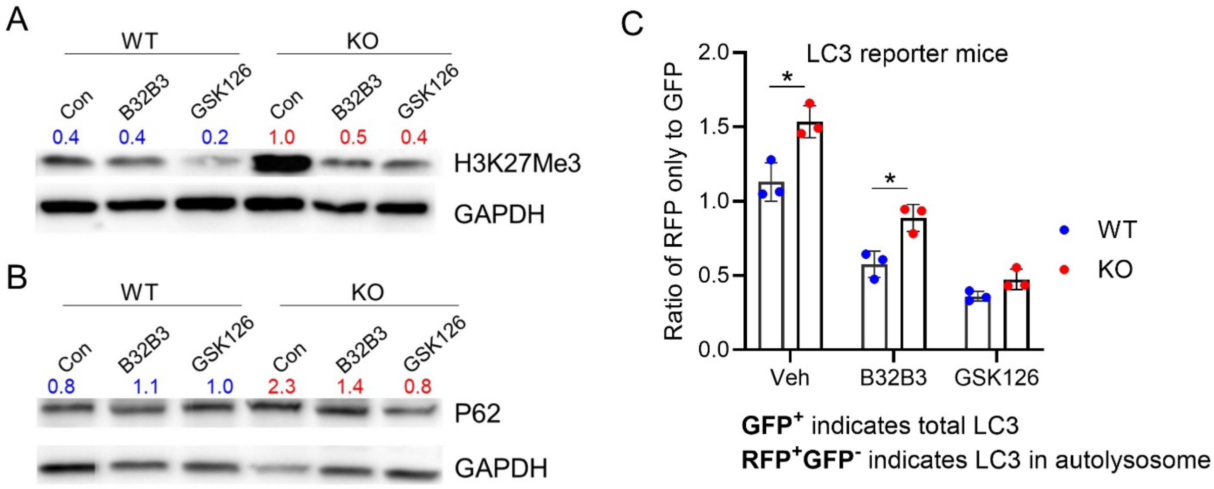


**Figure S9. Effects of VPRBP inhibitor B32B3 and EZH2 inhibitor GSK126 on CD8 T cells. (A-B)** WT and *Pim2* KO Pmel cells were active with gp100 peptide with DMSO, 1 µM B32B3 or 5 µM GSK126 for 3 days. H3K27Me3, P62 and GAPDH expression were examined by western blot. **(C)** *Pim2* KO mice on B6 background was crossed to LC3 reporter mice. T cells isolated from WT or *Pim2* KO LC3 reporter mice were simulated with plate-bounded anti-CD3/CD28 (2µg/ml) for 24h, and GFP and RFP were examined by flow. Bar graph shows the ratio of $RFP^+$ only (indicates LC3 in autolysosome) to $GFP^+$ (indicates total LC3 protein) cells. Data represent 2 independent experiments. Data were analyzed by one-way ANOVA and presented as mean ± SEM from technical replicates. *p<0.05, **p<0.01, ***p<0.001.

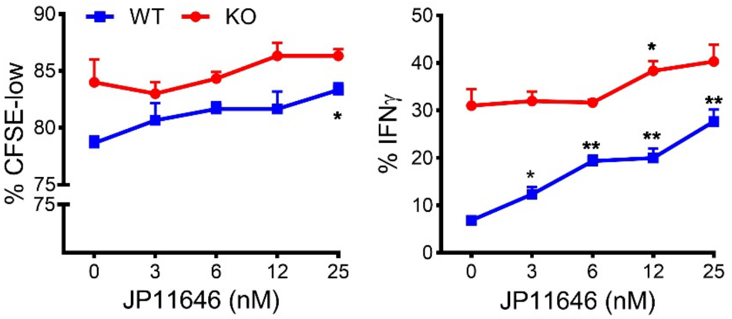


**Figure. S10. Effects of JP11646 on T-cell response to tumor specific antigen.** Splenocytes were isolated from WT or *Pim2* KO Clone 100 TCR-Tg mice, labeled with CFSE, and stimulated with 60 ng/ml $RNEU_{420-429}$ peptide in the presence of JP11646 at concentrations indicated. Cells were harvested 3 days after culture and measured for CFSE profile and intracellular IFNγ. Mean of $\%CFSE^{low}$ and $\%IFN\gamma^{+}$ cells in triplicated wells were shown on gated $CD8^{+}$ cells (mean ± 1SD). Data represents one of two replicate experiments. Data were analyzed by two-way ANOVA comparing individual concentration to no JP11646. Data is presented as mean ± SEM from technical replicates. *p<0.05, **p<0.01, ***p<0.001.

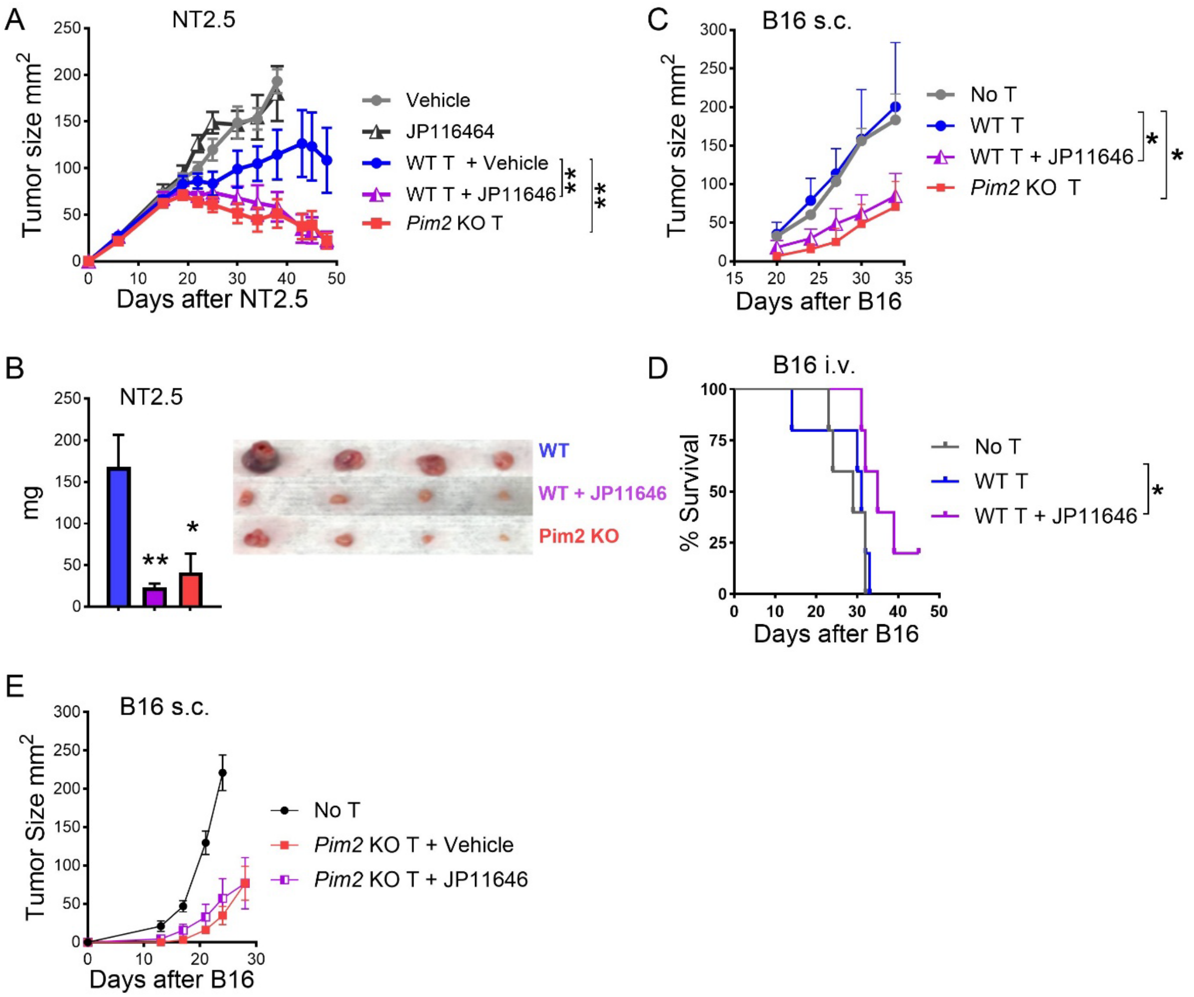


**Figure. S11. PIM2 inhibitor JP11646 improves efficacy of ACT. (A-B)** NT2.5 mammary cancer cells were injected under right mammary fat pads of $Thy1.1^+$ WT FVB mice. After tumor was established for 14 days, these tumor-bearing mice were adoptively transferred with $RNEU_{420-429}$ peptide pre-activated WT or *Pim2*$^{-/-}$ Clone 100 TCR-Tg CD8 T cells ($Thy1.2^+$). Following ACT, JP11646 or vehicle was administrated i.p. at 7.5 mg/kg twice a week for 4 weeks. **(A)** Tumor growth was monitored, and **(B)** tumors were isolated at the end of the experiment for weight measurement. Data represents one of two replicate experiments with n=10/group. **(C)** Ly5.1 B6 mice were s.c. injected with B16F10, followed with sublethal irradiation at 600cGy and adoptive transfer of gp100 peptide pre-activated WT or *Pim2* KO CD8 pmel T cells on day 7. Following ACT, JP11646 or vehicle was administrated i.p. at 7.5mg/kg twice a week for 4 weeks. Data

represents one of two replicate experiments with n=10/group. **(D)** Ly5.1 B6 mice were i.v. injected with B16F10, followed with sublethal irradiation at 600cGy and adoptive transfer of gp100 peptide pre-activated WT pmel CD8 T cells on day 3. Following ACT, JP11646 or vehicle was administrated i.p. at 7.5mg/kg twice a week for 4 weeks. Data represents one of two replicate experiments with n=5/group. **(E)** Ly5.1 B6 mice were s.c. injected with B16F10, followed with sublethal irradiation at 600cGy and adoptive transfer of gp100 peptide pre-activated *Pim2* KO CD8 pmel T cells on day 7. Following ACT, JP11646 or vehicle was administrated i.p. at 7.5mg/kg twice a week for 4 weeks. Data pooled from two replicate experiments with n=10/group. Data were analyzed by two-way ANOVA (A, C, E), one-way ANOVA (B) and log-rank test for survival curves (D). Data plotted are mean ± SEM from biological replicates. *p<0.05, **p<0.01, ***p<0.001.

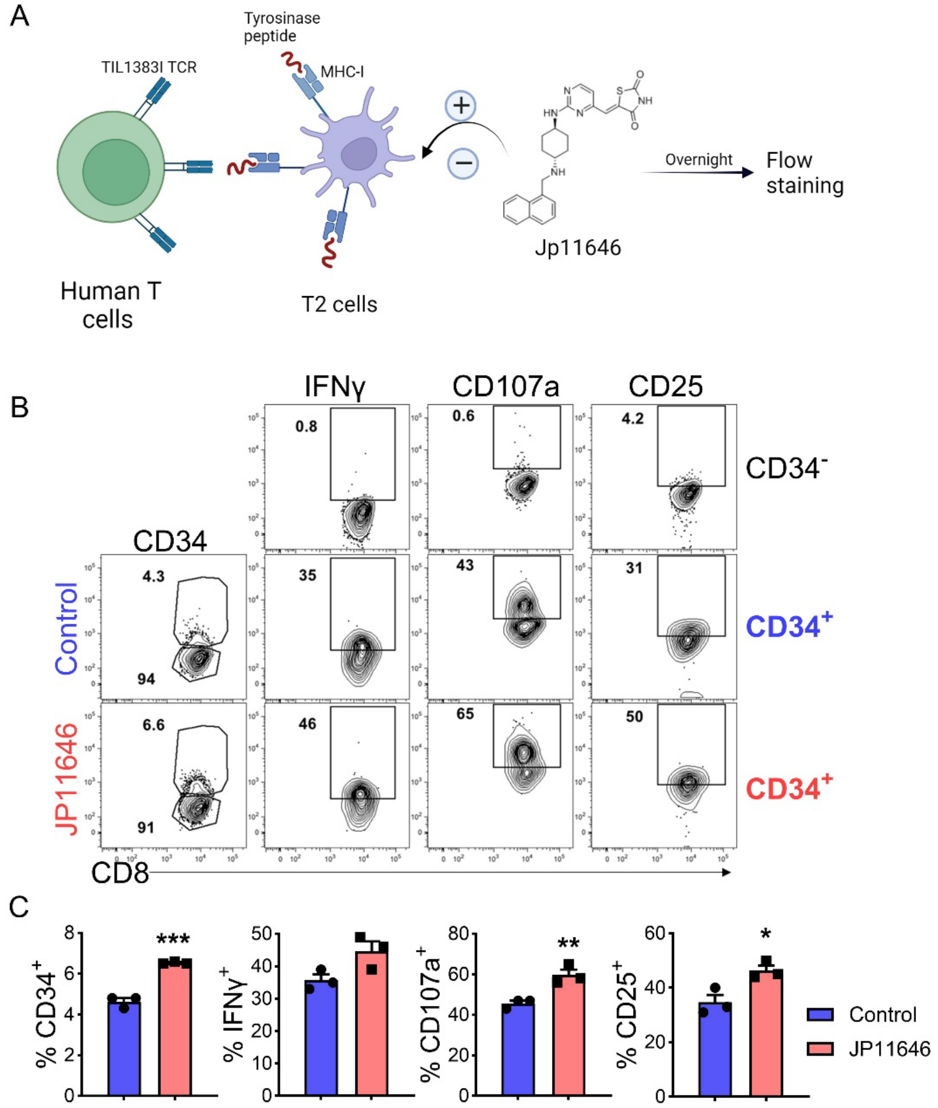


**Figure. S12. PIM2 inhibition increases effector differentiation of tumor-specific human T cells.** (A) Human T cells were transduced with lentivirus encoding the TIL1383I TCR and then cocultured with tyrosinase peptide pulsed T2 cells with DMSO or 12.5nM JP11646 overnight. Illusion was created with BioRender. (B) CD34 expression indicates TCR transduced T cells. The representative flow figures show IFNγ, CD107a, or CD25 expression in gated $CD34^+$ or $CD34^-$ CD8 T cells. (C) Bar graphs show % $CD34^+$ cells on gated CD8 T cells, and % $IFN\gamma^+$, $CD107a^+$, or $CD25^+$ on gated CD8+CD34+ cells. Data

represents one of two replicate experiments. Data were analyzed by two-tailed Student's t test and plotted as mean ± SEM from biological replicates. *p<0.05, **p<0.01, ***p<0.001.
