# Supplementary material for: Targeting PIM2 improves antitumor immunity through promoting effector function and persistence of CD8 T cells
Source: J Clin Invest. 2026 Jan 27;136(6):e192928. doi: 10.1172/JCI192928 (PMC12987625; doi:10.1172/JCI192928)
Supplement: Unedited blot and gel images [file jci-136-192928-s101.pdf]

Gel for Figure 6C

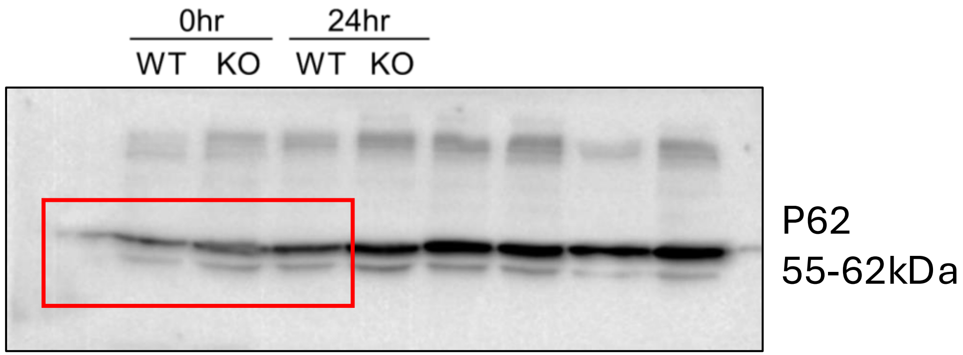

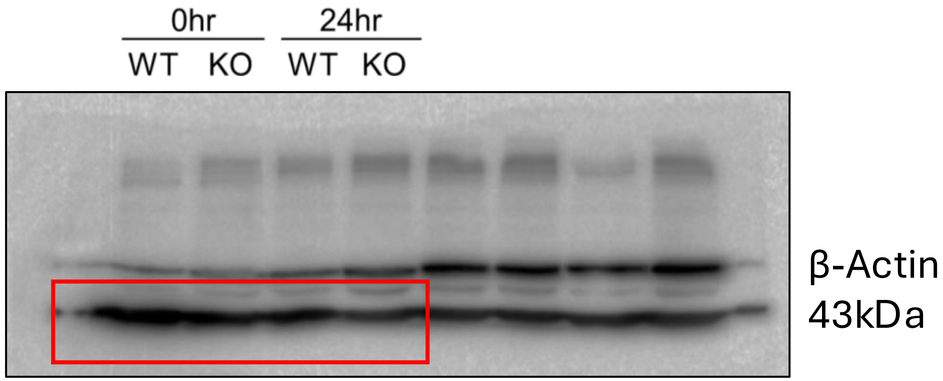

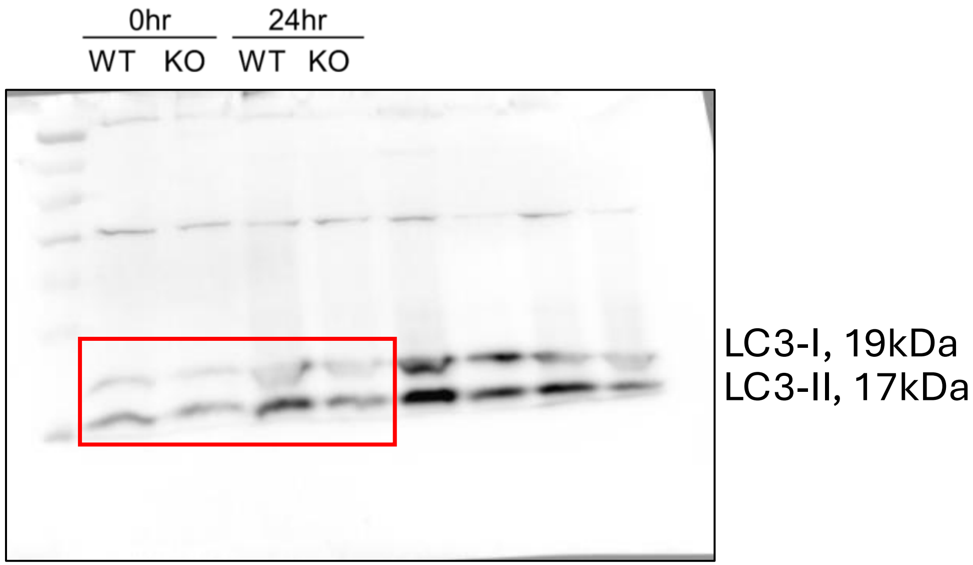

Gel for Figure 6E

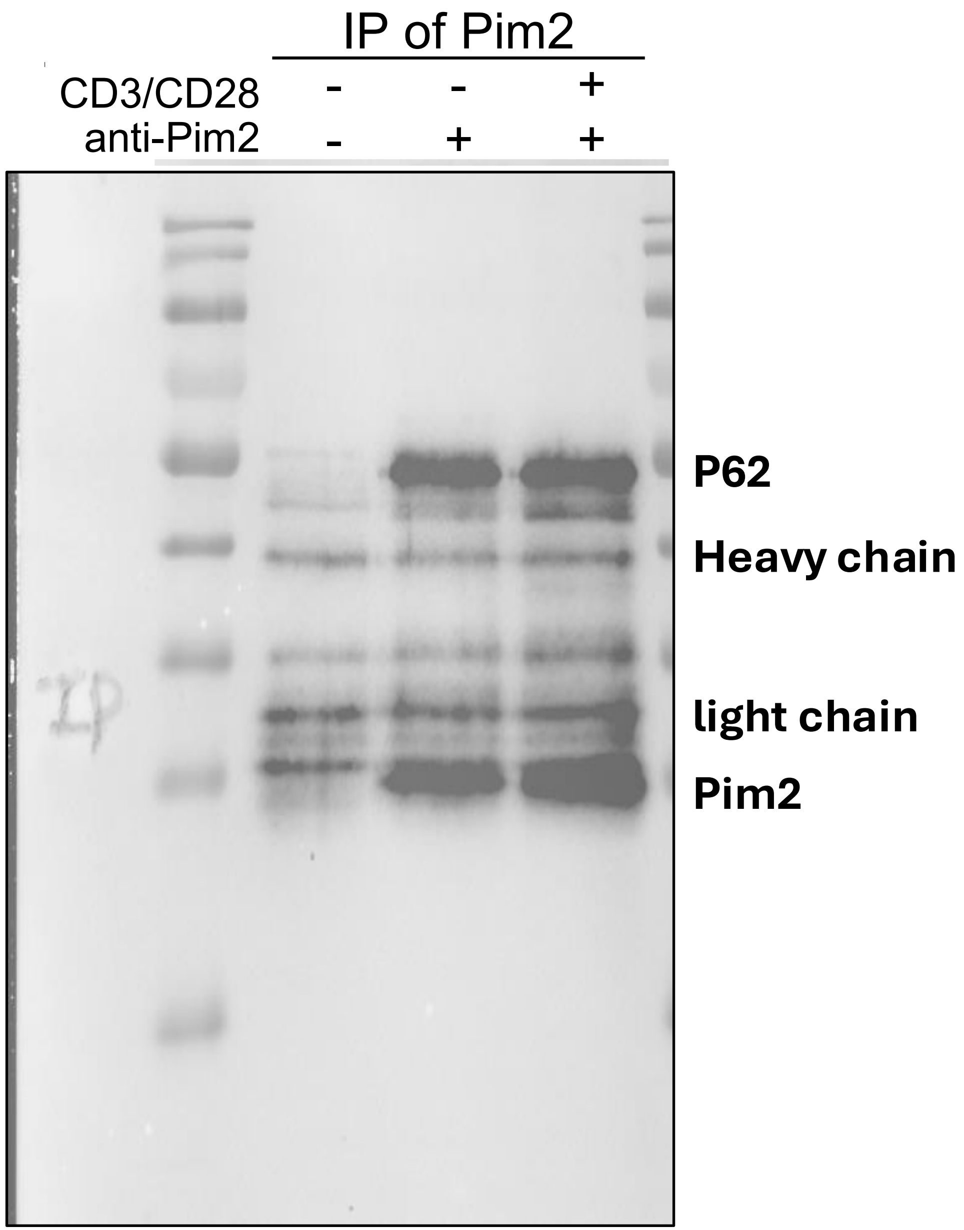

Gel for Figure 6G

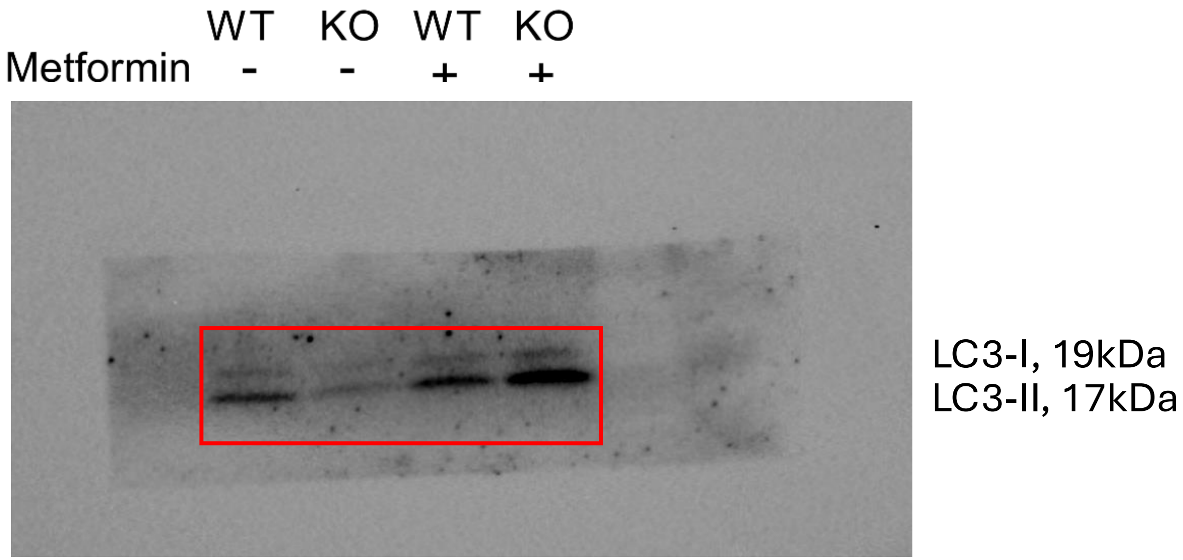

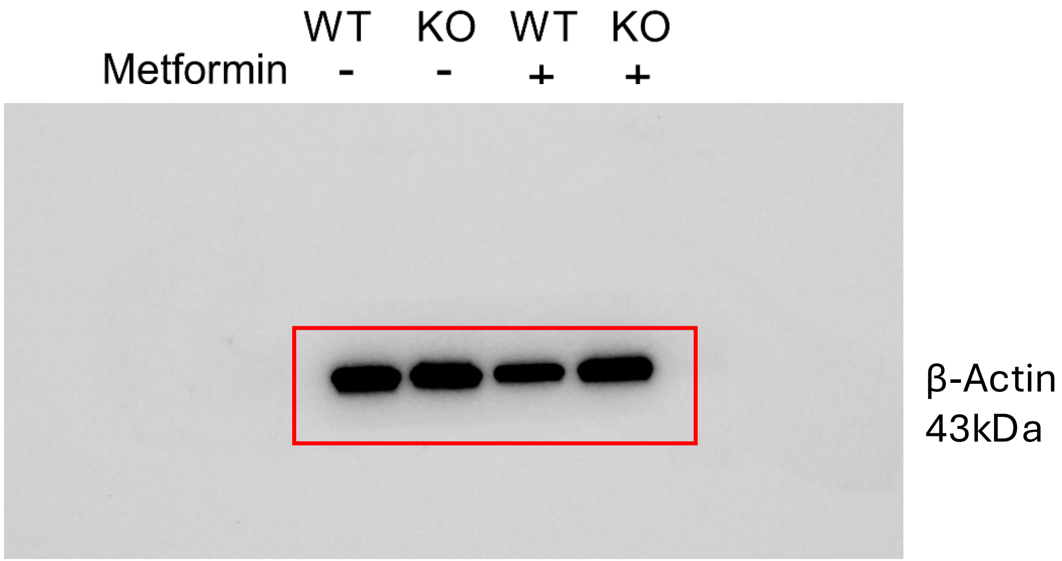

Gel for Figure 7D

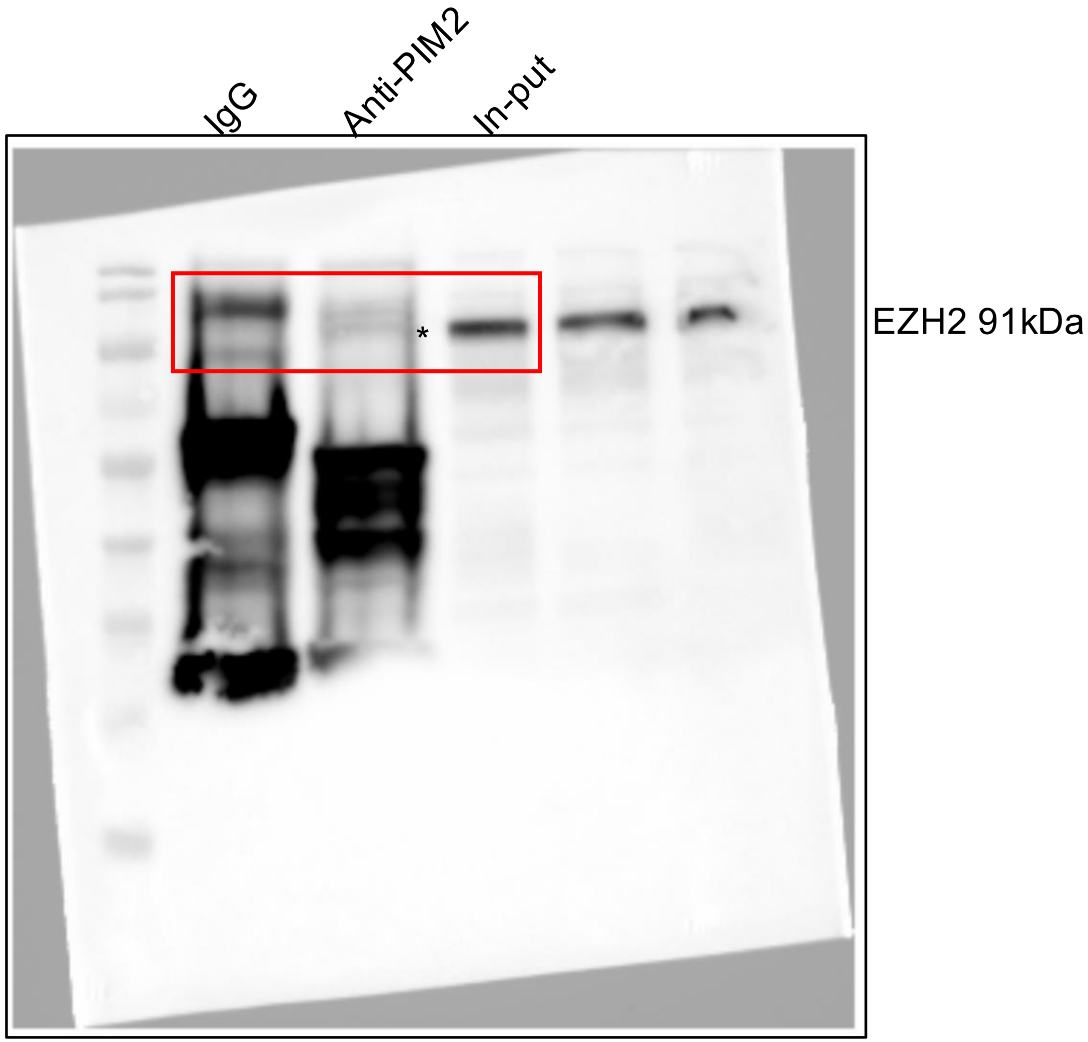

Gel for Figure 7D

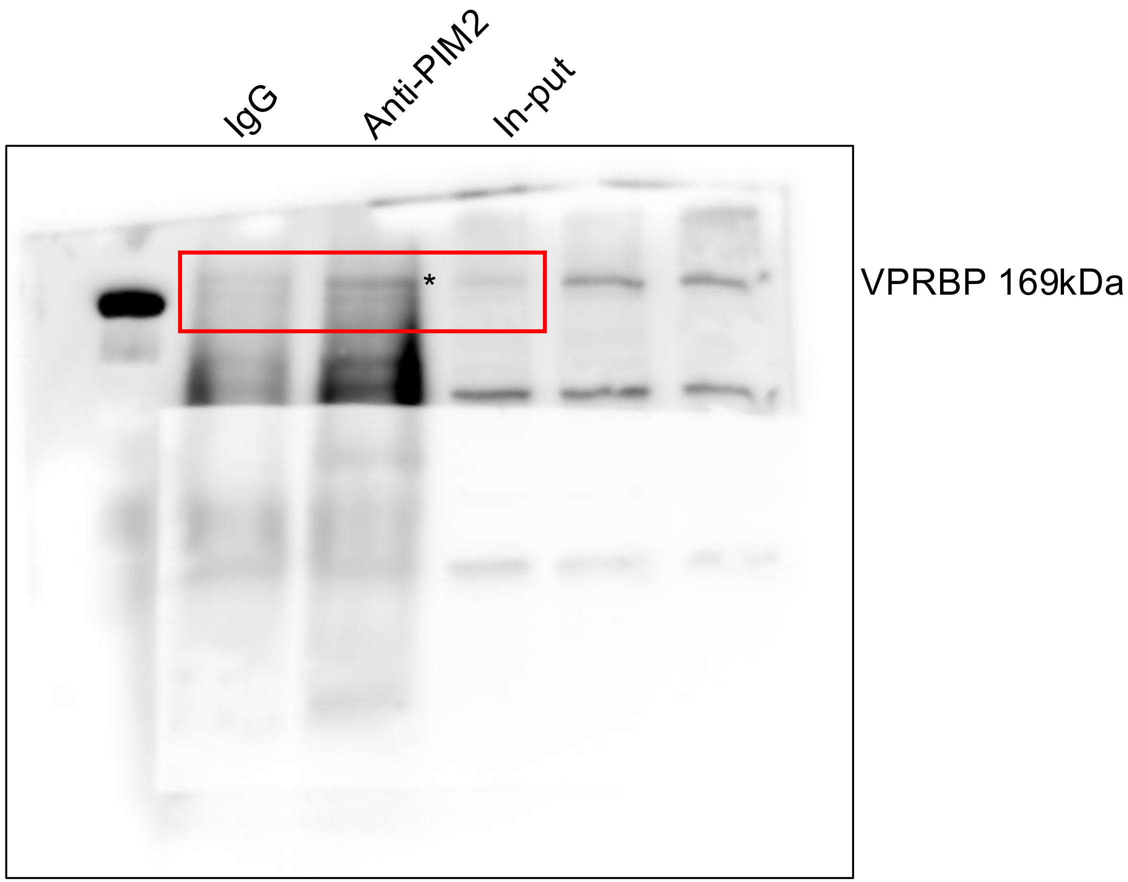

Gel for Figure 7D

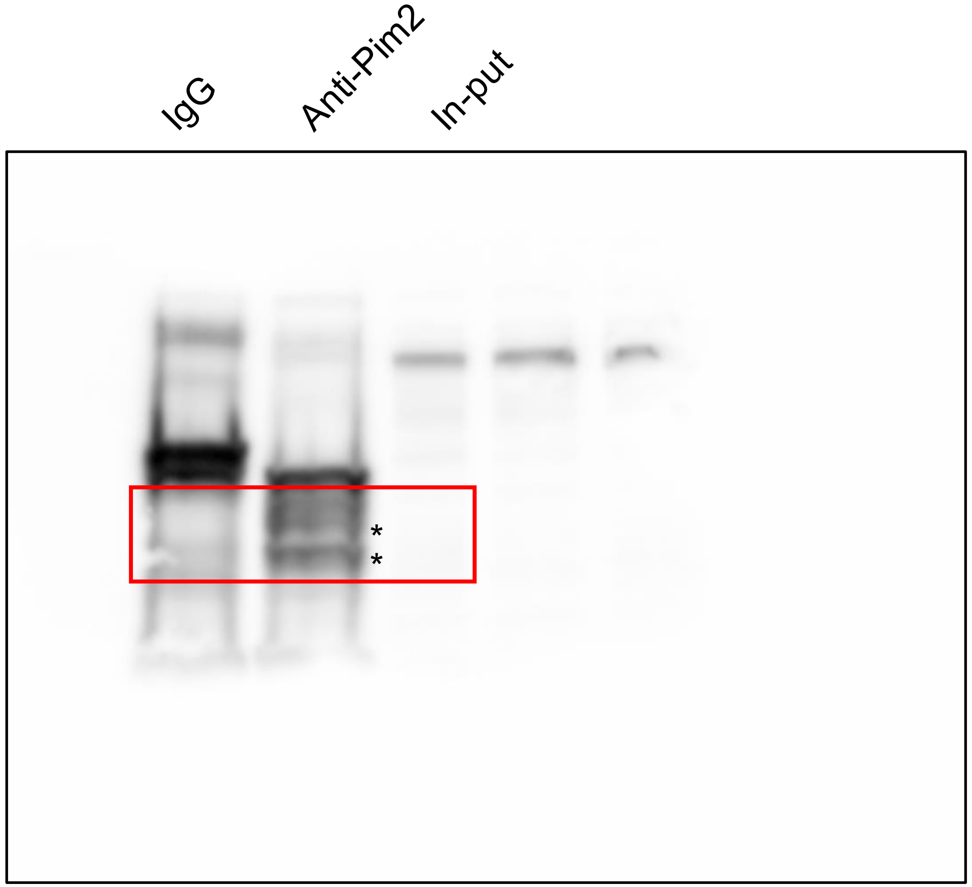

PIM2 34/38kDa

*

Gel for Figure 7E

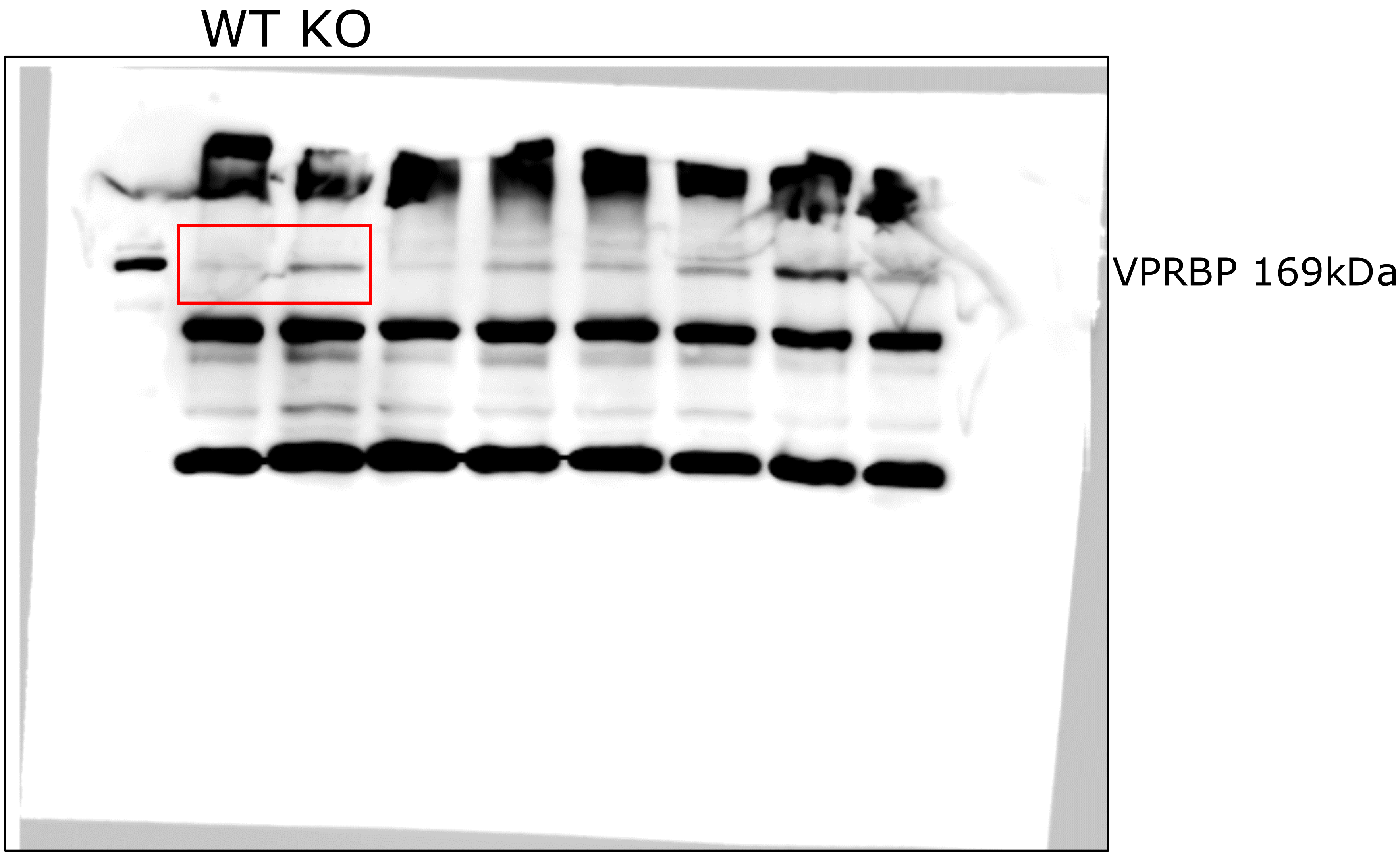

Gel for Figure 7E

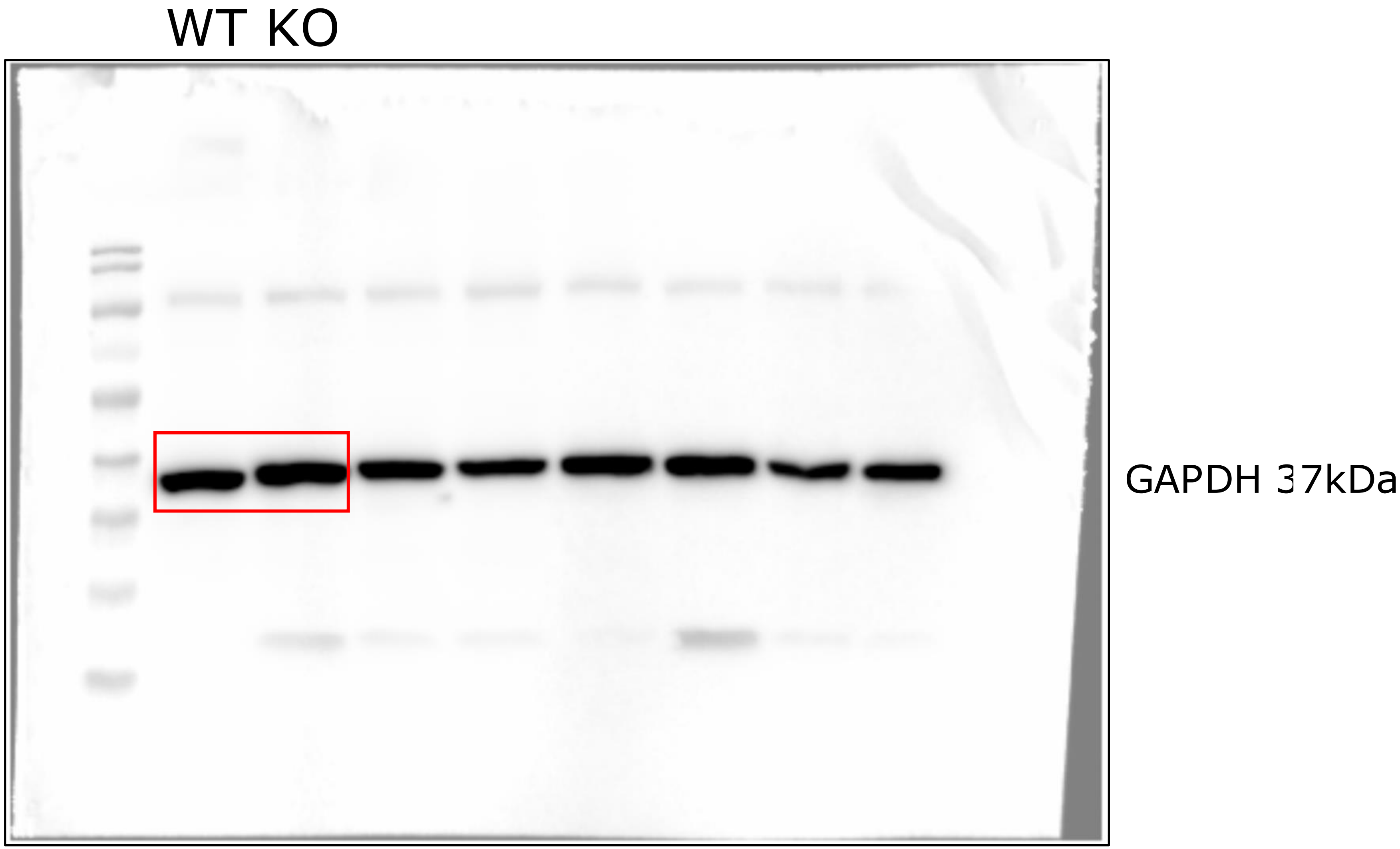

Gel for Figure 7F

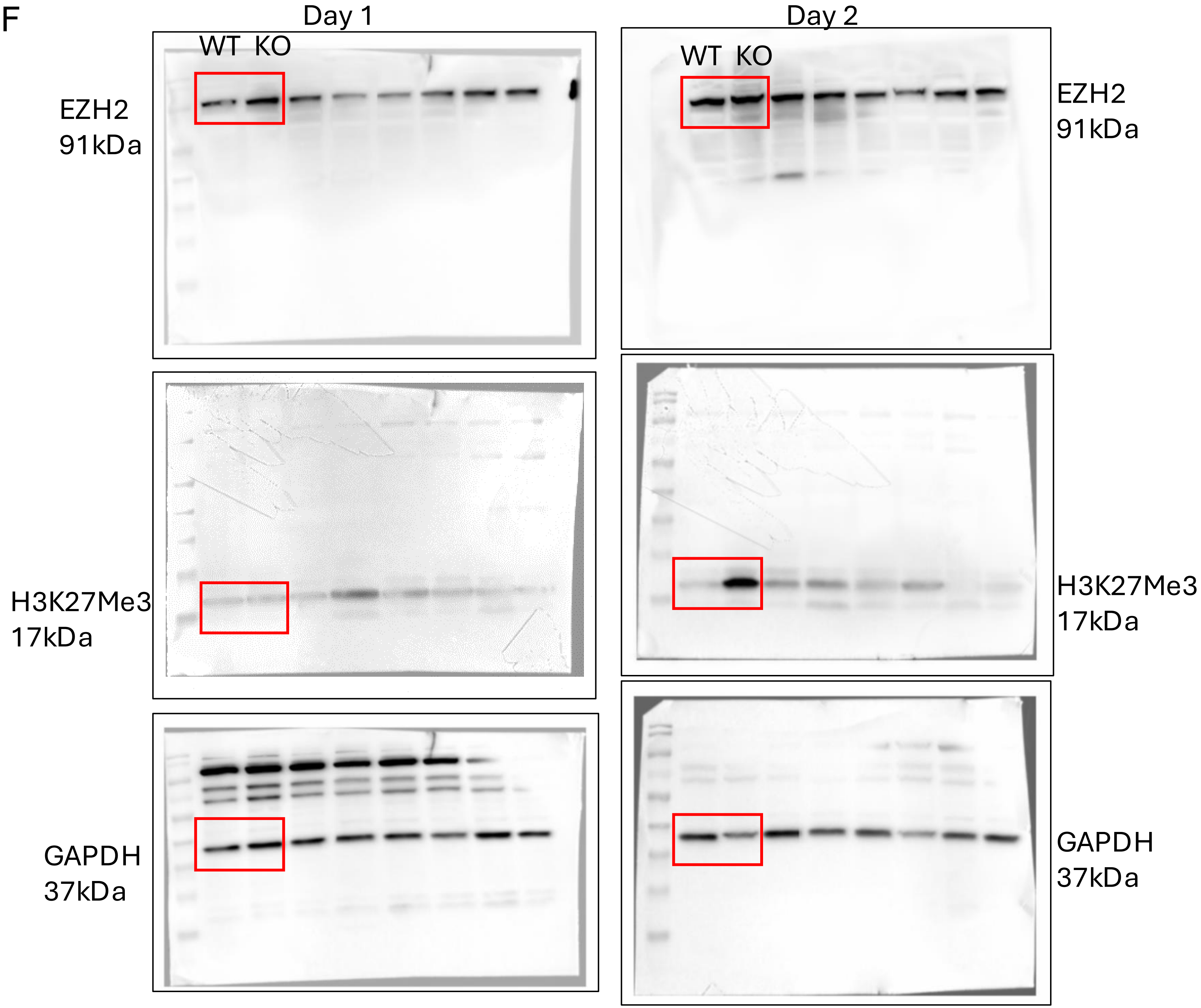

Gel for Figure 8A

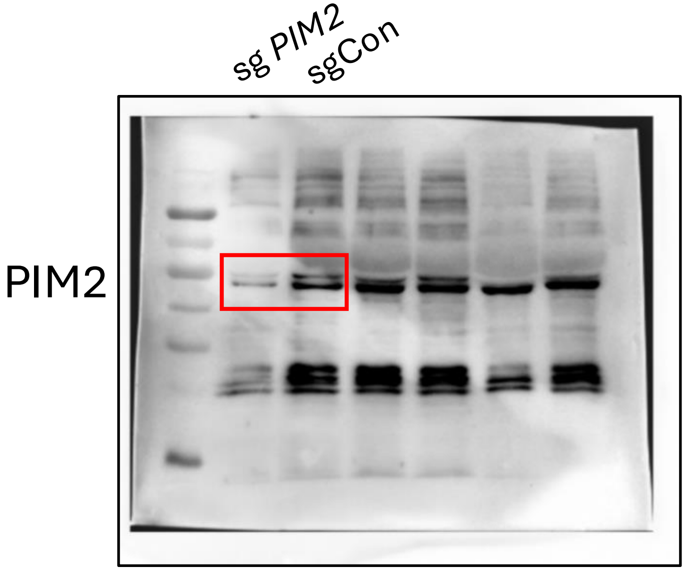

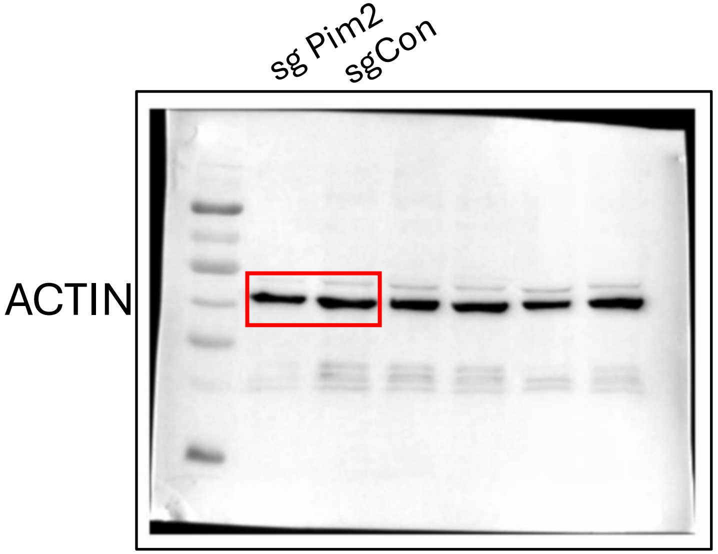

Gel for Figure S1B

PIM2
34kDa

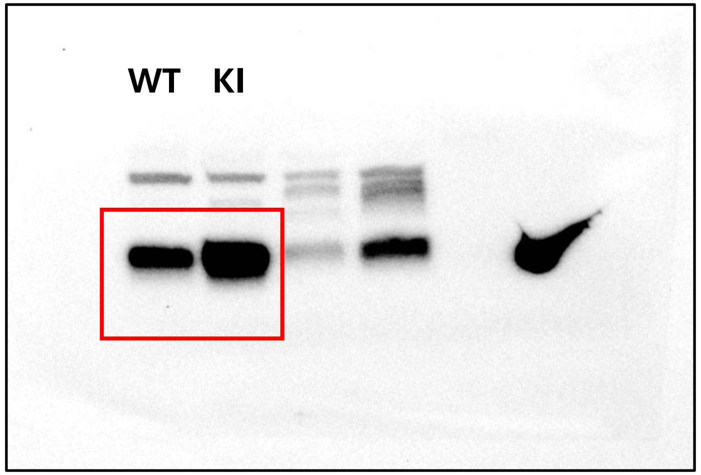

PIM2
34kDa

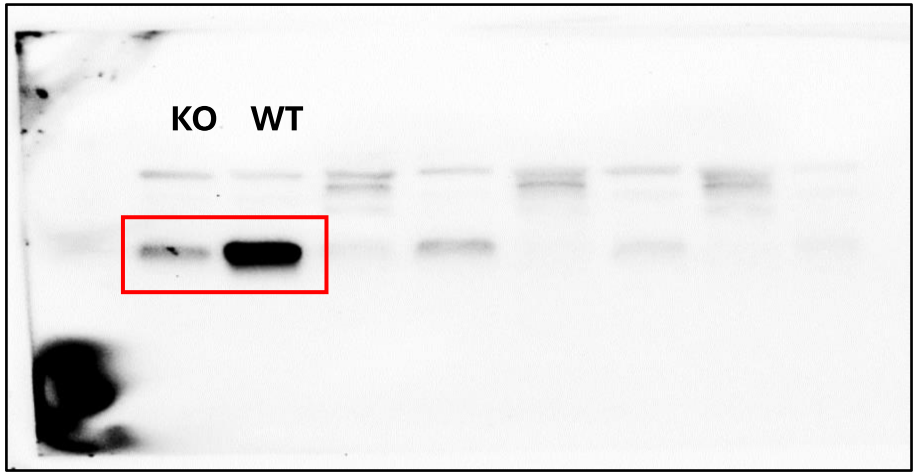

β-ACTIN
43kDa

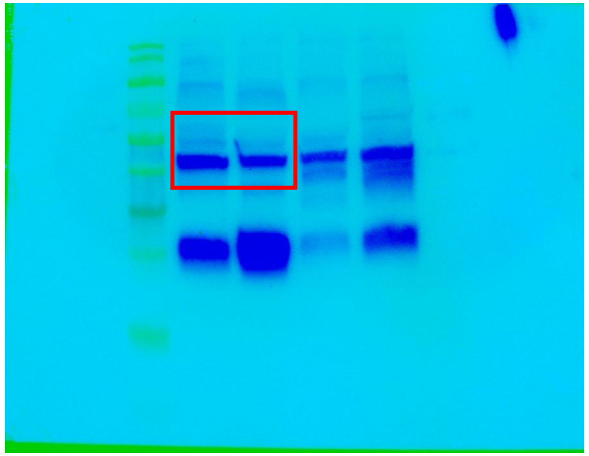

β-ACTIN
43kDa

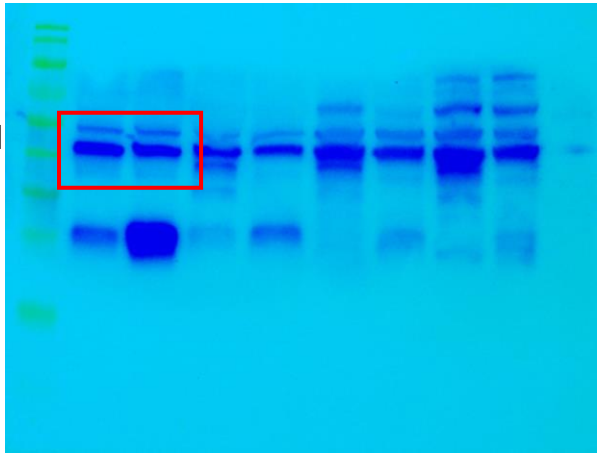

Gel for Figure S7A

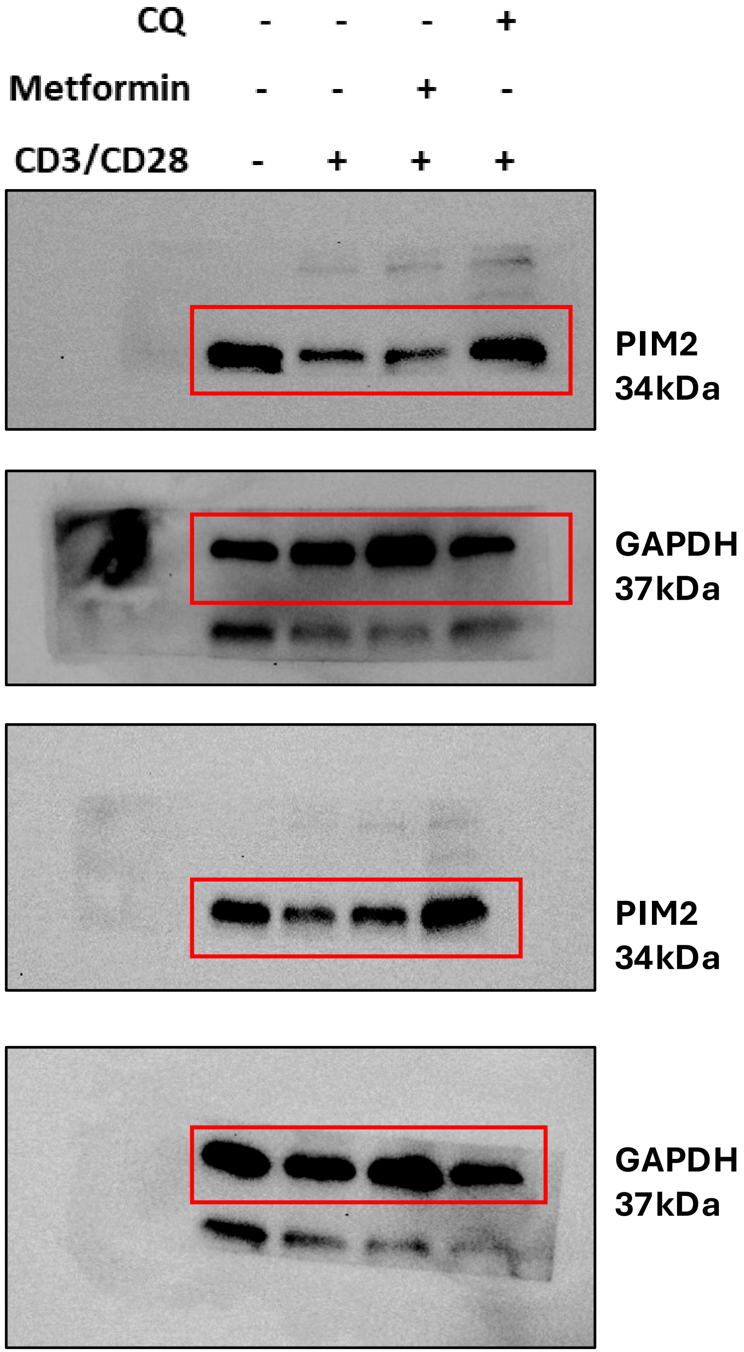

Gel for Figure S7B

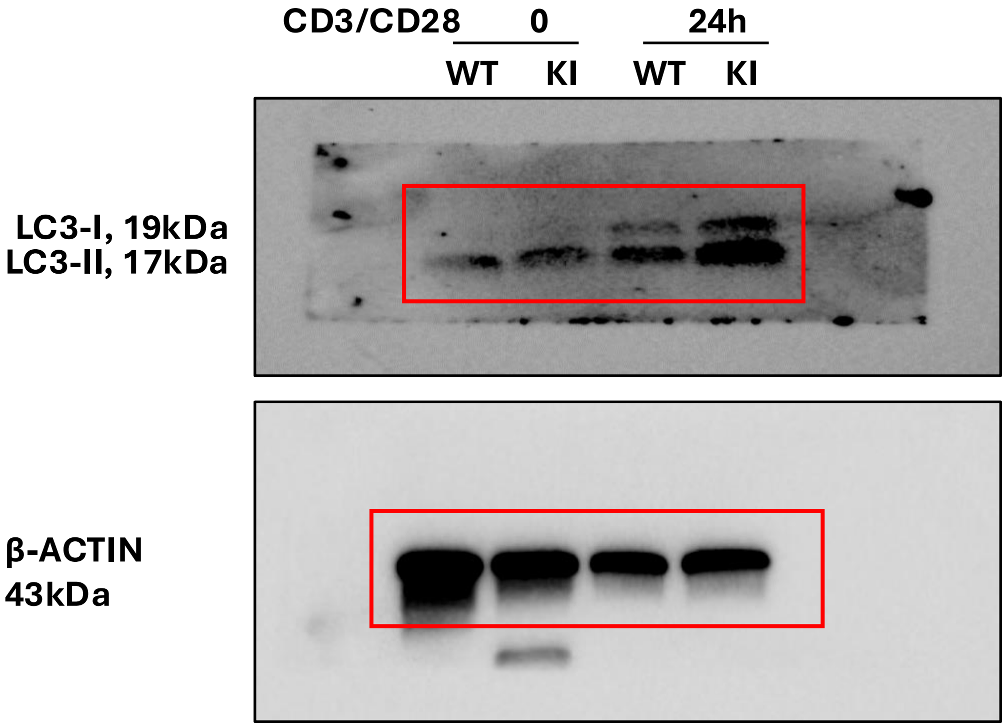

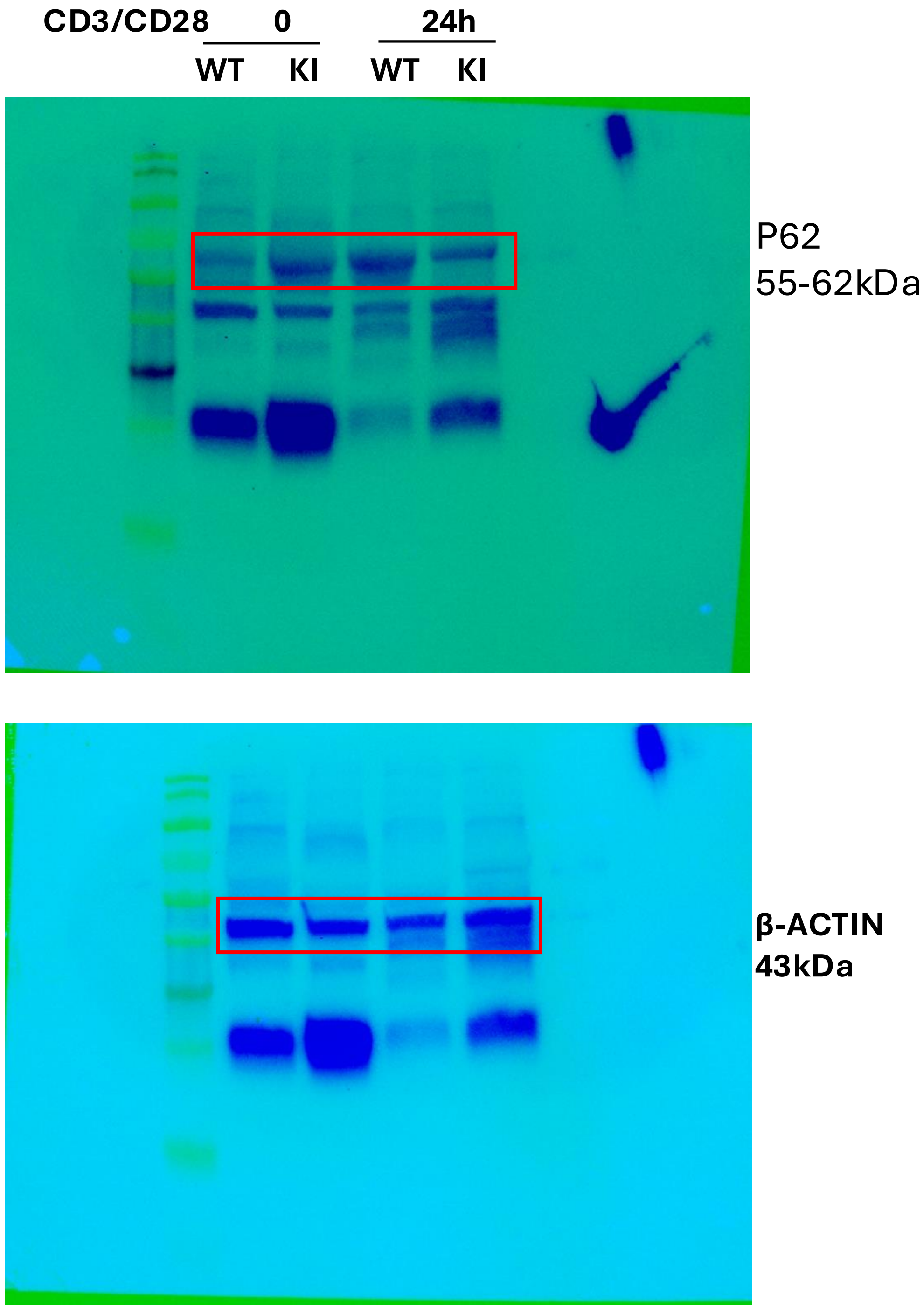

Gel for Figure S9A and B

H3K27Me3
17kDa

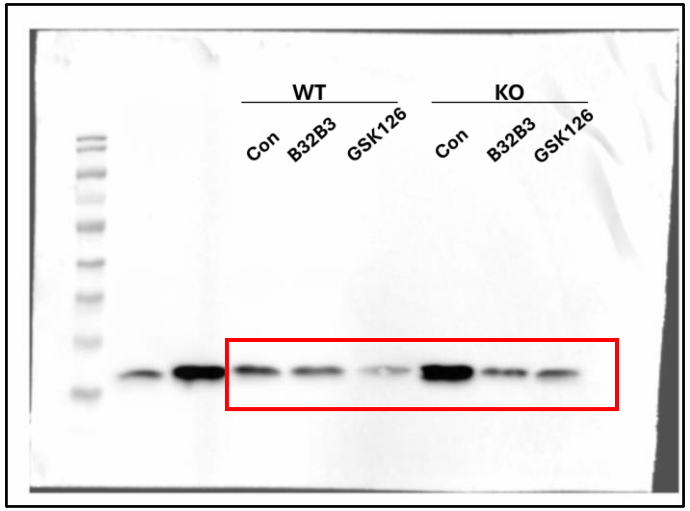

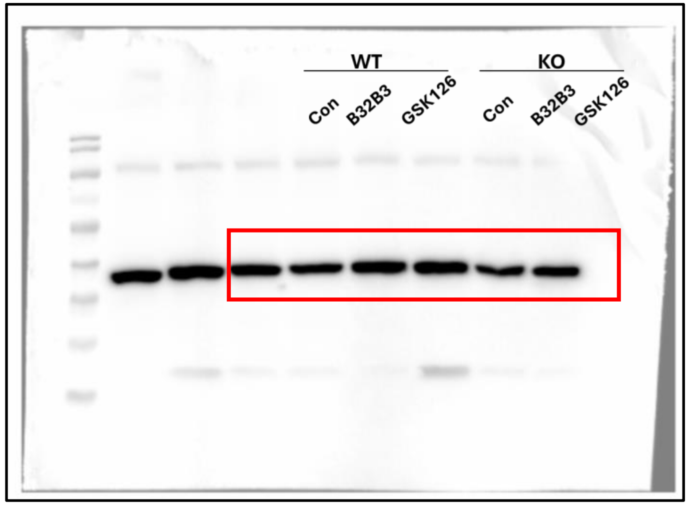

GAPDH 37kDa

P62
55-62kDa

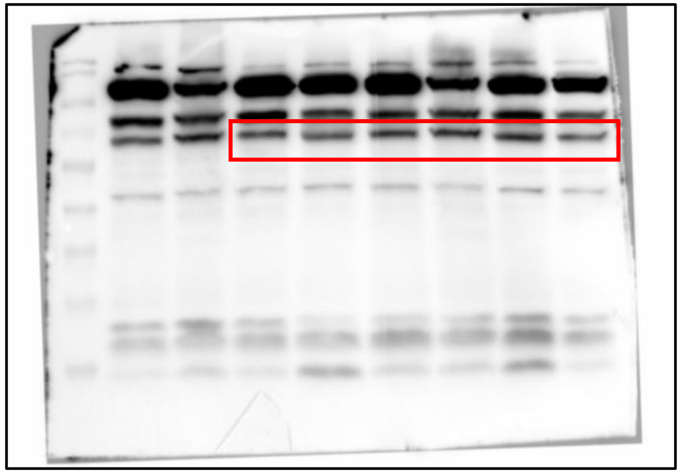

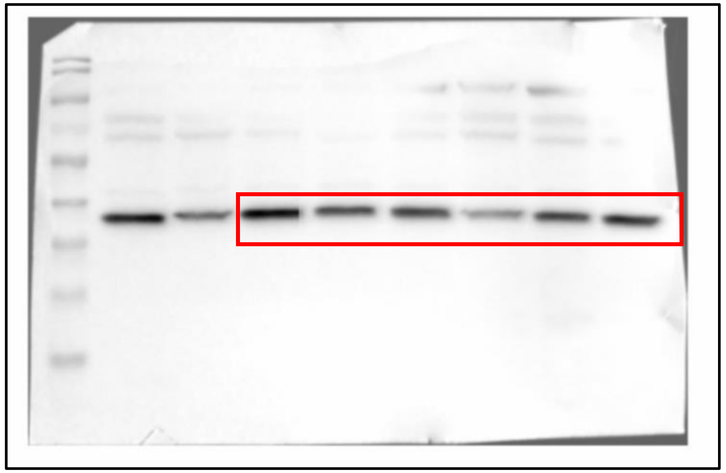

GAPDH 37kDa
